# Studying Trends of Auto-Regulation in Severe Head Injury in Paediatrics (STARSHIP): protocol to study cerebral autoregulation in a prospective multicentre observational research database study

**DOI:** 10.1136/bmjopen-2023-071800

**Published:** 2023-03-10

**Authors:** Shruti Agrawal, Michal M Placek, Deborah White, Esther Daubney, Manuel Cabeleira, Peter Smielewski, Marek Czosnyka, Adam Young, Suzanna Watson, Anna Maw, Peter John Hutchinson

**Affiliations:** 1 Department of Paediatrics, Cambridge University, Cambridge, UK; 2 Paediatric Intensive Care, Cambridge University Hospitals NHS Foundation Trust, Cambridge, UK; 3 Department of Clinical Neurosciences, University of Cambridge, Cambridge, UK; 4 Department of Mechanical Engineering, University College London, London, UK; 5 Paediatric Neuropsychology, Cambridge Centre for Paediatric Neuropsychological Rehabilitation, Cambridge, UK; 6 Centre for Child Development, Cambridge University Hospitals NHS Foundation Trust, Cambridge, UK; 7 Paediatric Neurologi, Cambridge University Hospitals NHS Foundation Trust, Cambridge, UK; 8 Academic Neurosurgery, University of Cambridge, Cambridge, UK

**Keywords:** paediatric intensive & critical care, physiology, neurological injury, trauma management

## Abstract

**Introduction:**

Studying cerebral autoregulation, particularly PRx (Pressure Reactivity Index), is commonly employed in adult traumatic brain injury (TBI) and gives real-time information about intracranial pathophysiology, which can help in patient management. Experience in paediatric TBI (PTBI) is limited to single-centre studies despite disproportionately higher incidence of morbidity and mortality in PTBI than in adult TBI.

**Project:**

We describe the protocol to study cerebral autoregulation using PRx in PTBI. The project called Studying Trends of Auto-Regulation in Severe Head Injury in Paediatrics is a multicentre prospective ethics approved research database study from 10 centres across the UK. Recruitment started in July 2018 with financial support from local/national charities (Action Medical Research for Children, UK).

**Methods and analysis:**

The first phase of the project is powered to detect optimal thresholds of PRx associated with favourable outcome in PTBI by recruiting 135 patients (initial target of 3 years which has changed to 5 years due to delays related to COVID-19 pandemic) from 10 centres in the UK with outcome follow-up to 1-year postictus. The secondary objectives are to characterise patterns of optimal cerebral perfusion pressure in PTBI and compare the fluctuations in these measured parameters with outcome. The goal is to create a comprehensive research database of a basic set of high-resolution (full waveforms resolution) neuromonitoring data in PTBI for scientific use.

**Ethics and dissemination:**

Favourable ethical approval has been provided by Health Research Authority, Southwest-Central Bristol Research Ethics Committee (Ref: 18/SW/0053). Results will be disseminated via publications in peer-reviewed medical journals and presentations at national and international conferences.

**Trial registration number:**

NCT05688462.

**STRENGTHS AND LIMITATIONS OF THIS STUDY**

- Studying Trends of Auto-Regulation in Severe Head Injury in Paediatrics is the first prospective multicentre cohort study in children powered to look at the association of cerebral autoregulation as studied by Pressure Reactivity Index with the neurodevelopmental outcome after severe traumatic brain injury (TBI).
- It will create a comprehensive research database of high-resolution neuromonitoring data with neurodevelopmental follow-up in paediatric TBI (PTBI) for scientific use.
- It will characterise patterns of optimal cerebral perfusion pressure in PTBI and compare the fluctuations in these measured parameters with outcome.

## Introduction

Traumatic brain injuries (TBIs) are a major burden to public health with a high incidence and potential long-term consequences.^1^ Incidence of TBI in children ranges from 47 to 280 per 100 000 and are a leading cause of hospital admission and death each year.^2^ Epidemiological studies have demonstrated that the incidence of hospitalisation, fatal brain injury and morbidity is disproportionately higher in children.^3 4^ Despite this, little is known about specific aspects of the paediatric TBI (PTBI) pathology, with most of the knowledge extrapolated from adults. Following head trauma, primary brain injury occurs as a result of the initial trauma. This triggers a cascade of secondary insults in hours to days following primary injury (eg, insufficient blood flow and oxygen supply, low blood pressure, brain swelling, increased intracranial pressure (ICP)), resulting in secondary brain injury. The secondary brain injury plays a significant role in the outcome. Therefore, the focus of standard management of PTBI in paediatric intensive care units (PICUs) is to limit these secondary brain insults.^5^ Current evidence and guidelines suggest use of ICP and cerebral perfusion pressure (CPP; defined as arterial blood pressure (ABP) minus ICP) measurements to guide therapies.^5^ Although there is some consensus on what level of ICP is unacceptably high, the optimal level of CPP is controversial in children. We and others have used advanced monitoring to investigate the association between CPP and clinical outcome^6–8^ and examined means of establishing individualised targets for CPP management, termed ‘CPP Optimal’ (CPPopt), based on analysis of the status of cerebral vascular reactivity, interpreted as a surrogate measure of autoregulation of cerebral blood flow (CBF). Interestingly, CPPopt shows significant variation from the guidelines recommendation, and CPPopt can be different between patients and change over time in each patient.^9 10^ This phenomenon is due to dysfunction and variability of autoregulatory mechanisms in patients with severe TBI that control CBF (and CPPopt).^11 12^ In adults and in our preliminary study in children, we found that the difference between CPP recommended by the guidelines and CPPopt was associated with worsened clinical outcome.^6 9 10^ For this reason, indices of cerebral autoregulation have been recommended to guide management of severe TBI in adults,^13^ but so far there is limited evidence to support their routine use in children.^7 8^

**Figure 1:**
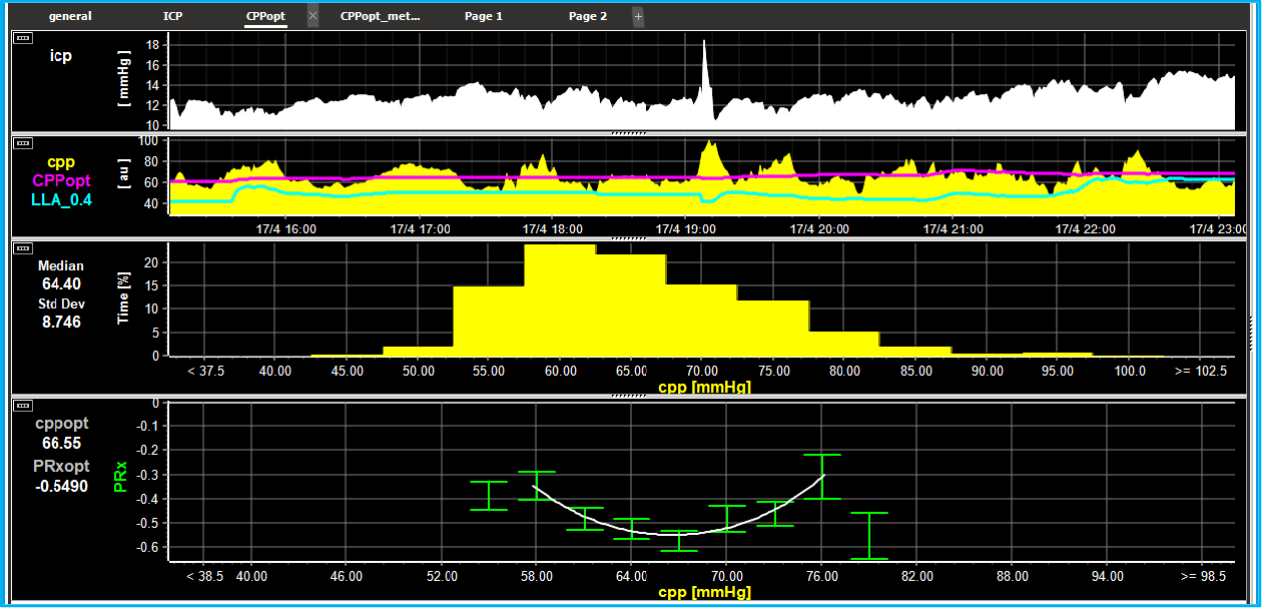
PRx and CPPopt calculation in real time in PTBI PRx and CPPopt calculation in real time in a patient recruited to STARSHIP in an 8-hour recording window. The top curve displays ICP in white followed CPP in yellow (CPPopt in the light grey line and lower limit of autoregulation in dark grey line). The curve at the bottom represen calculation of CPPopt. CPPopt is continuously calculated using a 5 min median CPP time trend alongside PRx. These PRx values are divided and averaged into CPP bins spanning 5 mm Hg. An automatic curve fitting method is applied to the binned data to determine the CPP value with the lowest associated PRx value. ABP, arterial blood pressure; CPP, cerebral perfusion pressure; CPPopt, optimum CPP; ICP, intracranial pressure; PRx, Pressure Reactivity Index; PTBI, paediatric traumatic brain injury; STARSHIP, Studying Trends of Auto-Regulation in Severe Head Injury in Paediatrics.

Normally, all severe PTBI in PICUs have continuous ICP and ABP monitoring; these values can be used to determine the state of cerebrovascular autoregulation and subsequently CPPopt. In health, fluctuations in systemic circulation are compensated by reactive changes in cerebral vasomotor tone to maintain steady CBF. For example, a drop in ABP induces vasodilatation, which increases cerebral blood volume and ICP. Conversely, an increase in ABP leads to vasoconstriction, which decreases cerebral blood volume and ICP. This relationship can be studied continuously in real time through the correlation of fluctuations in ABP and ICP waveforms as the cerebrovascular Pressure Reactivity Index (PRx).^14^ This index can range from −1 to +1, with negative or 0 values indicating intact pressure reactivity and normal autoregulation (associated with better clinical outcome), while positive values indicating disturbed pressure reactivity and abnormal autoregulation (associated with poorer outcome).^12^ PRx has been found useful prognostic marker in adult patients with TBI.^14–16^ Plotting PRx against measured CPP can determine the CPP at which the vasculature is most reactive, therefore, estimating CPPopt for an individual patient at a particular point in time (figure 1). To date, apart from our experience,^6^ only two other small paediatric data sets have been published about PRx and CPPopt.^7 8^ This study aims to provide vital information on optimal thresholds of PRx and CPP in children to limit secondary brain injury and improve outcomes.

### Study aim

We aim to create a research database of high-resolution monitoring data from PTBI patients along with the clinical and demographic details and neuropsychological outcome up to 12 months postictus, from 10 UK PICUs to be used for research purposes. As the first step, we will analyse the data to determine retrospective optimal clinical parameters when managing these patients. Our hypothesis is that we can define the optimal parameters for CPP based on indices of autoregulation (PRx) and that the optimal parameters of CPP and PRx in PTBI will differ to adults, both qualitatively and quantitatively.

### Study objectives

Primary objective: To detect thresholds of PRx associated with favourable outcome in PTBI.

Secondary objectives:

1. Characterise patterns of CPPopt in PTBI as a function of age, severity and character of injury, ICP, etc.
2. Compare the fluctuations in these measured parameters with the outcome.
3. Establish a comprehensive paediatric database of a basic set of high resolution (pulse waveforms resolution) neuromonitoring data including ICP, ABP and ECG.

## Methods and analysis

This is an observational prospective cohort study. Children admitted to ten identified PICUs in the UK with severe TBI with a clinical need for ICP and ABP monitoring will be included for data collection. The database will contain routinely monitored physiological parameters during management of PTBI in PICU. Without disturbing the routine care and management of these patients, we aim to collect this data continuously in a bedside laptop equipped with the study software (ICM+, Cambridge Enterprise, Cambridge, UK). Alongside these physiological parameters, we will also collect relevant clinical and demographic data (details below) and follow these patients for up to 12 months post ictus to assess neuropsychological outcome. The created database will be used to analyse the state of cerebral autoregulation. We will also analyse the data to see if we can derive CPPopt based on the state of autoregulation and compare it with the patient outcome at 6 and 12 months. The database can also be subsequently used in the future projects testing hypotheses inspired by the experience gained in the adult TBI population research and to answer important questions about severe PTBI.

### Patient and public involvement

We work closely with the Children’s Brain Injury Trust and have a child and family worker embedded in the team; they have given their input from patients and public perspective into the study protocol and design, which has been acceptable to patients and families.

The patients are not being directly consented for the study as the children and young people who are eligible for the study are not able to give meaningful informed consent or assent from the very nature of the disease making them eligible for inclusion to the study. The data are only shared and stored after consent from the patient’s family or legal guardians; the consent is also sought for the follow-up. Although we will not be able to tell the results to individual patients, the results will be published in peer-reviewed journals. Since this is not an interventional study, there is no research burden to an individual patient.

### Inclusion criteria

Patients 16 years old or younger at the time of injury, admitted to PICU for management of TBI with TBI-related pathology confirmed on CT or MRI and clinical requirement for invasive monitoring of ICP and ABP, will be eligible for recruitment. There are no exclusion criteria for the project.

### Patient recruitment and consent

Each PTBI patient admitted to the recruiting PICU with invasive monitoring of ICP and ABP is considered for recruitment. The research team at each recruiting PICU identifies the patients and sets up high-resolution data collection to the project laptop under an anonymised ID as soon as possible after the patient has been commenced on invasive monitoring and stabilised on PICU. Physiological and clinical data are recorded under this unique study ID. As the acute phase of the project is purely observational and does not involve any therapeutic intervention or change in regular standard care, we take a deferred consent for acute data collection and sharing. This is important to obtain a bias-free sample and ensure capture of early changes in intracranial physiology after head injury. When appropriate, the identified guardian/parent is informed and consented for data sharing and follow-up before the patient is discharged from the hospital. For patients who die before the consent can be obtained, the clinician in charge of patient’s clinical care only (not study team) will access and anonymise data of the deceased patient under his/her clinical care; this anonymised data will then be provided to the study team.

### Data collection

ICP will be monitored with an intraparenchymal microsensor inserted into the right (predominately but can be left) frontal cortex and ABP will be monitored in the radial or femoral artery with a zero calibration at the level of the right atrium. The head end elevation is part of the standard international protocol for management for PTBI, and any deviations from this policy in individual cases will be recorded. Full resolution data of routinely monitored physiological parameters in managing PTBI (ICP, ABP, ETCO_2_, SpO_2_ and ECG) will be collected from bed-side monitors. Pulsatile signals (ICP, ABP and ECG) will be recorded with the maximum sampling frequency offered by the bed-side monitors, at least 100 Hz for invasive pressures, and at least 200 Hz for ECG. This will be done using relevant, monitor type specific, data export protocols implemented in the study (ICM+) software running on a bed-side laptop. The same software will be later used for the retrospective analysis of all stored signals. The continuous data collection will be stopped when the patient either starts to wake up or dies and the invasive monitoring is stopped. Apart from general demographics and clinical data, the other data being collected are mechanism and details of injury, relevant history, postresuscitation Glasgow coma scale, initial pupillary size and reactivity, prehospital hypoxia/hypotension/hyperthermia/cardiopulmonary resuscitation, injury and disease severity scores (Paediatric Index of Mortality II score, Injury Severity Score), CT scan findings. We are also collecting: daily laboratory values (haemoglobin, glucose, sodium, lactate), PaO_2_, PaCO_2_ and FiO_2_, PEEP, medical interventions for TBI (positioning, collar, temperature, CO_2_, glucose, hypertonic saline or mannitol, sedation/paralysis, barbiturate coma, anti-seizure medication, use of vasoactive drugs to maintain blood pressure and surgical interventions (haematoma evacuation, elevation of depressed fracture, CSF diversion-external ventricular drainage, new brain imaging, any other relevant event.

### Data management and monitoring

Each patient is allocated a unique study ID and all the collected data are anonymised to this ID. Appointed data manager is responsible for monitoring data transfer to Cambridge, for data verification, validation and repackaging, and storing it in the archive location on the server. Full ethical and legal practice is being followed and database will be managed securely under the supervision of data custodian. The project is funded for 5 years (30 June 2023) at present and we will endeavour to get further funding to carry on the data collection and database further in the near future. The data will be stored on a secure server located on a private network behind a tight firewall at the sponsoring institute. We will use the already established system in use for similar larger adult project, CENTER-TBI (https://www.center-tbi.eu/). Briefly, all the physiological time series data collected in each participating site using ICM+ software are stripped of any identifiers and packaged into the HDF5 (https://www.hdfgroup.org/) based format. The file is then uploaded using the Secure Shell Network (SSH) data transfer protocol function of ICM+ to the Cambridge server using public/private key authentication and the username/password distributed in an encrypted format and installed on the ICM+ laptop. The data comes into ‘upload’ folder on that server and is then promptly moved to a staging folder, inaccessible via the SSH connection. The data are then scrutinised for potential problems, artefacts, stripped of any potential identifiers embedded into the annotations, stripped of the data recording date (the timestamps are recorded as relative to the date of ictus, not kept with the data file) and archived permanently. The original data are then removed. All analyses are subsequently done on that preprocessed data. The data are backed up daily, using incremental backup procedure.

### Outcome measures

The primary outcome will be measured by Glasgow Outcome Scale Extended Paediatric version (GOSE-Peds) at 12-month postinjury against the PRx.

The study will also include the following secondary outcome measures:

- GOSE-Peds at 6 months against the PRx.
- Delta CPP (CPP deviation from CPPopt) against the GOSE-Peds at 6 and 12 months.
- Rate of severe TBI-related surgical interventions undertaken during the index admission.
- Mortality (30 days, 6 months 12 months).
- Length of stay in hospital and rehabilitation.

There is more detailed neuropsychology assessment at 12 months follow-up by using telephone-based interviews and postal questionnaires with parents by using the following tests (child versions will be used where available): Behaviour Rating Inventory for Executive Function (BRIEF), Pediatric Quality of Life Inventory (PedsQL), Strengths and Difficulties Questionnaire (SDQ), Children’s Communication Checklist (CCC), Child and Adolescent Scale of Participation (CASP) and Revised Children’s Anxiety and Depression Scale (RCADS). In addition, with parental permission, school teacher will be contacted for SDQ and BRIEF with postal questionnaires.

Due to the covid-related impact on research, to circumvent the missed follow-ups for the recruited patients:

1. Where possible, site teams will carry out telephone interview with parents/legal guardian to ascertain child’s neurodevelopmental performance at the first anniversary of child’s head injury for retrospective GOSE-Peds score at 12 months. There is precedence to this approach, especially in children where the developmental milestones/neurodevelopment are judged from memory based on how an individual child performed at different time points in their developmental life.^17–20^
2. If the child was seen in Acquired Brain Injury/Neurology/Neuro-psychology/Neurodevelopmental clinic at a specific site, the local research team will review the clinic letters/notes to ascertain child’s outcome as close as possible to 12 months from the head injury.

### Statistics

#### Power calculation

The sample size has been calculated assuming a mean (±SD) PRx of 0.03±0.13 in patients with favourable outcome and a mean (±SD) PRx of 0.10±0.17 (SD) for patients with unfavourable outcome (from our previous work).^6^ For a one-sided analysis, using a favourable/unfavourable ratio of 0.77, with 80% power and alpha error of 5%, and allowing for losses (protocol violations, withdrawal of consent or lost to follow-up) the study will require 135 patients. Recruiting 135 patients with severe TBI in 36 months should have been feasible as based on the national audits of PICU (PICAnet report 2015: 188, 210, 211 patients in 2013, 2014 and 2015, respectively, with ICP devices from the 10 UK centres recruiting into the current study) and major trauma (TARN report on 2 years of severe injury in children (2013–2014): 1018 children in 2 years with severe head injury) with an estimated recruitment rate of 5–7 patients per site per year. On the basis of hospital episode statistics and data from the ongoing local severe TBI audit, approximately 5–10 patients with severe TBI are admitted in a medium-sized PICU each year.

However, due to the COVID-19 pandemic, the recruitment has been slow (pause of non-COVID-19 research projects, research staff redeployment to clinical work and lockdowns leading to changes in trauma patterns in children), and the study has been extended until December 2024 in agreement with the sponsor and funding organisation.

#### Statistical analysis

Continuous variables will be assessed for normality and expressed as mean (SD) or median (IQR), depending on the underlying distribution of the data. Categorical variables will be reported as counts and proportions. Differences in physiological values between survivors and non-survivors will be interrogated with the Mann-Whitney U test for non-normally distributed continuous variables, Student’s t-test for normally distributed continuous variables and Fisher’s exact test for categorical variables. All tests will be two tailed and unadjusted for multiple comparisons. Spearman rank correlation coefficient (ρ) will be used to see correlations between variables. Identification of critical threshold values of PRx for predicting unfavourable outcome will be performed using iterative χ^2^ tests, as per the method described by Sorrentino *et al*.^21^ To account for any possible confounding effect of the differing periods of monitoring and day post admission on which monitoring starts on outcome associations, ordinal logistic regression analysis will be performed. The independent effect of each continuously monitored variable on GOSE-Peds will be assessed by including the length of monitoring and monitoring commencement day post admission as covariates in the regression models. Where appropriate PRx will be Fisher transformed to remove the <−1; 1> constrain and ensure normalisation of its distribution. The effect of time on PRx will be assessed using repeated-measures analysis of variance with each patient treated as a random effect.

### Participant confidentiality and data protection

The data collection part of this research study is limited to accessing, collecting and analysing information that is monitored and placed in the medical record as part of the child’s standard care while being treated for severe TBI in PICU. Our plan to protect patient-subject identifiers from improper use and disclosure is consistent with our plans to protect the overall data of the study. Specifically, access to patient-subject identifiers will be limited to study personnel that require its use for clinical reasons. The anonymised data will be used by the investigators and research staff listed on this application which will help ensure that there is no improper use or disclosure. Second, a limited amount of personal information (specifically, only the month and year of birth which is essential to establishing the age of the subject) will be collected. Third, all patient-subject identifiers will be kept in a locked cabinet within the recruiting hospital, which can only be accessed by the study staff on the delegation log. Fourth, all subjects within the study will be given a unique, de-identified study ID and this study ID will be utilised for importing data to the study database. We believe that our plan adequately ensures that patient-subject identifiers will be protected from improper use and disclosure. There will be no patient identifiable information shared outside the respective clinical teams looking after each patient. Within each individual centre, the patient identifiable information will not be seen/shared with anyone who does not access it otherwise (outside the clinical teams). Within the centres, the consent forms and the data collection will be stored in a secure locked cupboard within a secure locked room. The individual centres will be instructed to save the data for 5 years (to ensure data auditing and clearance), after which time the collected identifiable data will be destroyed).

The project is to be carried out in conformation with the spirit and the letter of the Declaration of Helsinki, and in accord with the ICH Good Clinical Practice Guidelines. The application, the database management and monitoring have full support from the patient safety department at sponsoring institute, and the protocol has been approved by them. The entire project will be conducted under close scrutiny of data custodian and the patient safety department.

## Ethics approval and regulatory considerations

Ethical approval was provided by Health Research Authority, South West-Central Bristol Research Ethics Committee (Ref: 18/SW/0053). As the project has approval for research database, it did not require site-specific ethics. The project was reviewed and approved at each participating site before starting recruitment. The study protocol, patient information sheet, consent forms and other study-related documents were reviewed and approved by the REC with regard to their scientific content and compliance with applicable research regulations involving human subjects. The current study protocol is V5 dated 28 August 2018. The study is registered at ClinicalTrails.gov with ID—NCT05688462.

### Dissemination

The results will be published in peer-reviewed journals and presented at relevant national and international conferences and seminars.

## Supplementary Material

Reviewer comments

Author's manuscript
