# Supplementary material for: Studying Trends of Auto-Regulation in Severe Head Injury in Paediatrics (STARSHIP): protocol to study cerebral autoregulation in a prospective multicentre observational research database study
Source: BMJ Open. 2023 Mar 10;13(3):e071800. doi: 10.1136/bmjopen-2023-071800 (PMC10008199; doi:10.1136/bmjopen-2023-071800)
Supplement: Author's manuscript [file bmjopen-2023-071800.draft_revisions.pdf]

BMJ Open is committed to open peer review. As part of this commitment we make the peer review history of every article we publish publicly available.

When an article is published we post the peer reviewers' comments and the authors' responses online. We also post the versions of the paper that were used during peer review. These are the versions that the peer review comments apply to.

The versions of the paper that follow are the versions that were submitted during the peer review process. They are not the versions of record or the final published versions. They should not be cited or distributed as the published version of this manuscript.

BMJ Open is an open access journal and the full, final, typeset and author-corrected version of record of the manuscript is available on our site with no access controls, subscription charges or pay-per-view fees (http://bmjopen.bmj.com).

If you have any questions on BMJ Open's open peer review process please email info.bmjopen@bmj.com

BMJ Open

Studying Trends of Auto-Regulation in Severe Head Injury in Paediatrics (STARSHIP): Protocol to study cerebral autoregulation in a prospective multicentre observational research database study.

Journal:	*BMJ Open*
Manuscript ID	bmjopen-2023-071800
Article Type:	Protocol
Date Submitted by the Author:	11-Jan-2023

Complete List of Authors:	Agrawal, Shruti; Cambridge University Hospitals NHS Foundation Trust, Paediatric Intensive Care; Cambridge University Hospitals<br>Placek, Michal M.; University of Cambridge, Department of Clinical Neurosciences<br>White, Deborah; Cambridge University Hospitals NHS Foundation Trust, Paediatric Intensive Care<br>Daubney, Esther; Cambridge University Hospitals NHS Foundation Trust, Paediatric Intensive Care<br>Cabeleira, Manuel; University College London<br>Smielewski, Peter; University of Cambridge, Clinical Neuroscience<br>Czosnyka, Marek; University of Cambridge,<br>Young, Adam; University of Cambridge, Department of Clinical Neurosciences<br>Watson, Suzanna; Cambridge Centre for Paediatric Neuropsychological Rehabilitation, Paediatric Neuropsychology; Cambridge University Hospitals NHS Foundation Trust, Centre for Child Development<br>Maw, Anna; Cambridge University Hospitals NHS Foundation Trust, Paediatric Neurologi<br>Hutchinson, Peter; University of Cambridge, Academic Neurosurgery

Keywords:	Paediatric intensive & critical care < PAEDIATRICS, Physiology < NATURAL SCIENCE DISCIPLINES, Neurological injury < NEUROLOGY, TRAUMA MANAGEMENT

SCHOLARONE™
Manuscripts

Studying Trends of Auto-Regulation in Severe Head Injury in Paediatrics (STARSHIP): Protocol to study cerebral autoregulation in a prospective multicentre observational research database study.

Shruti Agrawal MD[1,2], Michal Placek PhD[3], Deborah White MSc[1], Esther Daubney BSc[1], Manuel Cabeleira MSc[4], Peter Smielewski PhD[3], Marek Czosnyka PhD[3,5], Adam Young PhD[3], Suzanna Watson PhD[6,7], Anna Maw FRCPCH[7], Peter J Hutchinson PhD, FMedSci[3]

1. Paediatric Intensive Care, Cambridge University Hospitals, Cambridge, UK
2. Department of Paediatrics, University of Cambridge, UK
3. Division of Neurosurgery, Department of Academic Neurosciences, University of Cambridge UK
4. Department of Mechanical Engineering, University College London, UK
5. ISE, Warsaw University of Technology, Poland
6. Cambridge Centre for Paediatric Neuropsychological Rehabilitation, Cambridge, UK
7. Centre for Child Development, Paediatric Neurology, Cambridge University Hospitals, Cambridge, UK

Corresponding Author:

Dr Shruti Agrawal

PICU, Level 3, Box 7

Addenbrookes Hospital

Hills Road, Cambridge CB2 0QQ

Email: shruti.agrawal1@nhs.net, sa882@cam.ac.uk

Ph: +441223348066

Word Count: 3086

Trial Registration: Clinicaltrials.gov awaited.

For peer review only

Abstract:

Introduction: Studying cerebral autoregulation, particularly PRx (pressure reactivity index), is commonly employed in adult traumatic brain injury (TBI) and gives real-time information about intracranial pathophysiology which can help in patient management. Experience in paediatric TBI (PTBI) is limited to single centre studies despite disproportionately higher incidence of morbidity and mortality in PTBI than in adult TBI.

Project: We describe the protocol to study cerebral autoregulation using PRx in PTBI. The project called STARSHIP (**S**tudying **T**rends of **A**uto-**R**egulation in **S**evere **H**ead **I**njury in **P**aediatrics) is a multi-centre prospective ethics approved research database study from 10 centres across the UK. Recruitment started in July 2018 with financial support from local/national charities (Action Medical Research for Children, UK).

Methods: The first phase of the project is powered to detect optimal thresholds of PRx associated with favourable outcome in PTBI by recruiting 135 patients (initial target of 3 years which has changed to 5 years due to delays related to Covid-19 pandemic) from 10 centres in the UK with outcome follow-up to one year post ictus. The secondary objectives are to characterize patterns of optimal cerebral perfusion (CPPopt) in PTBI and compare the fluctuations in these measured parameters with outcome.

Implications: The goal is to create a comprehensive research database of a basic set of high-resolution (full waveforms resolution) neuro-monitoring data in PTBI for scientific use.

Ethics and Dissemination: Favourable ethical approval has been provided by Health Research Authority, Southwest-Central Bristol Research Ethics Committee (Ref: 18/SW/0053). Results will be disseminated via publications in peer reviewed medical journals and presentations at national and international conferences.

Key words: Paediatric Traumatic Brain Injury, children, cerebral autoregulation, pressure reactivity index (PRx), optimal cerebral perfusion pressure.

Strengths and Limitations:

- STARSHIP is the first prospective multi-centre cohort study in children powered to look at the association of cerebral autoregulation as studied by PRx with the neuro-developmental outcome after severe TBI.
- It will also create a comprehensive research database of a basic set of high-resolution (full waveforms resolution) neuro-monitoring data in PTBI for scientific use.

For peer review only - http://bmjopen.bmj.com/site/about/guidelines.xhtml

- It will also characterize patterns of optimal cerebral perfusion (CPPopt) in PTBI and compare the fluctuations in these measured parameters with outcome.
- The results from this study will help in planning future research to individualise treatment targets for PTBI.
- The delays to study completion and loss of follow-ups due to Covid-19 related pandemic may affect the study outcomes.

For peer review only

For peer review only - http://bmjopen.bmj.com/site/about/guidelines.xhtml

Introduction: Traumatic brain injuries (TBI) are a major burden to public health with a high incidence and potential long term consequences (1). Incidence of TBI in children ranges from 47 to 280 per 100,000 and are a leading cause of hospital admission and death each year (2). Epidemiological studies have demonstrated that the incidence of hospitalisation, fatal brain injury, and morbidity is disproportionately higher in children (3,4). Despite this, little is known about specific aspects of the paediatric TBI (PTBI) pathology, with most of the knowledge extrapolated from adults. Following head trauma, primary brain injury occurs as a result of the initial trauma. This triggers a cascade of secondary insults in hours to days following primary injury (for e.g., insufficient blood flow and oxygen supply, low blood pressure, brain swelling, increased intracranial pressure, etc.), resulting in secondary brain injury. The secondary brain injury plays significant role in outcome. Therefore, the focus of standard management of PTBI in Paediatric Intensive Care Units (PICU) is to limit these secondary brain insults (5). Current evidence and guidelines suggest use of intracranial pressure (ICP) and cerebral perfusion pressure [CPP; defined as arterial blood pressure (ABP) minus ICP] measurements to guide therapies (5). Although there is some consensus on what level of ICP is unacceptably high, the optimal level of CPP is controversial in children. We and others have used advanced monitoring to investigate the association between CPP and clinical outcome (6-8) and examined means of establishing individualised targets for CPP management, termed 'CPP Optimal' (CPPopt), based on analysis of the status of cerebral vascular reactivity, interpreted as a surrogate measure of autoregulation of cerebral blood flow (CBF). Interestingly, CPPopt shows significant variation from the guidelines recommendation, and CPPopt can be different between patients and change over time in each patient (9,10). This phenomenon is due to dysfunction and variability of autoregulatory mechanisms in patients with severe TBI that control cerebral blood flow (and CPPopt) (11, 12). In adults and in our preliminary study in children, we found that the difference between CPP recommended by the guidelines and CPPopt was associated with worsened clinical outcome (6,9,10). For this reason, indices of cerebral autoregulation have been recommended to guide management of severe TBI in adults (13), but so far there is limited evidence to support their routine use in children (7,8).

Normally, all severe PTBI in PICUs have continuous ICP and ABP monitoring; these values can be used to determine state of cerebrovascular autoregulation and subsequently CPPopt. In health, fluctuations in systemic circulation are compensated by reactive changes in cerebral vasomotor tone to maintain steady cerebral blood flow. For example, a drop in ABP induces vasodilatation, which increases cerebral blood volume and ICP. Conversely, an increase in ABP leads to vasoconstriction, which decreases cerebral blood volume and ICP. This relationship can be studied continuously in real time through the correlation of fluctuations in ABP and ICP

For peer review only - http://bmjopen.bmj.com/site/about/guidelines.xhtml

waveforms as the cerebrovascular pressure reactivity index (PRx) (14). This index can range from −1 to +1, with negative or zero values indicating intact pressure reactivity and normal autoregulation (associated with better clinical outcome), while positive values indicating disturbed pressure reactivity and abnormal autoregulation (associated with poorer outcome) (12). PRx has been found useful prognostic marker in adult TBI patients (14-16). Plotting PRx against measured CPP can determine the CPP at which the vasculature is most reactive, therefore estimating CPPopt for an individual patient at a particular point in time (Figure 1). To date, apart from our experience (6), only two other small paediatric data sets have been published about PRx and CPPopt (7,8). This study aims to provide vital information on optimal thresholds of PRx and CPP in children to limit secondary brain injury and improve outcomes.

Study Aim: We aim to create a research database of high-resolution monitoring data from PTBI patients along with the clinical and demographic details and neuro-psychological outcome up to 12 months post-ictus, from 10 UK PICUs to be used for research purposes. As the first step, we will analyze the data to determine retrospective optimal clinical parameters when managing these patients. Our hypothesis is that we can define the optimal parameters for CPP based on indices of autoregulation (PRx) and that the optimal parameters of CPP and PRx in PTBI will differ to adults, both qualitatively and quantitatively.

Study Objectives:

Primary Objective: To detect thresholds of PRx associated with favourable outcome in PTBI.

Secondary objectives:

1. Characterise patterns of optimal CPP in PTBI as a function of age, severity and character of injury, intracranial pressure, etc.
2. Compare the fluctuations in these measured parameters with the outcome.
3. Establish a comprehensive paediatric database of a basic set of high resolution (pulse waveforms resolution) neuro-monitoring data including ICP, ABP and ECG.

Methods and Analysis: This is an observational prospective cohort study. Children admitted to ten identified PICUs in the UK with severe TBI with a clinical need for ICP and ABP monitoring will be included for data collection. The database will contain routinely monitored physiological parameters during management of PTBI in PICU. Without disturbing the routine care and management of these patients, we aim to collect this data

For peer review only - http://bmjopen.bmj.com/site/about/guidelines.xhtml

continuously in a bedside laptop equipped with the study software (ICM+, Cambridge Enterprise Ltd, Cambridge, UK). Alongside these physiological parameters, we will also collect relevant clinical and demographic data (details below) and follow these patients for up to 12 months post ictus to assess neuro-psychological outcome. The created database will be used to analyze the state of cerebral autoregulation. We will also analyze the data to see if we can derive optimal cerebral perfusion pressures based on the state of autoregulation and compare it with the patient outcome at 6 and 12 months. The database can also be subsequently used in the future projects testing hypotheses inspired by the experience gained in the adult TBI population research & to answer important questions about severe PTBI.

Inclusion Criteria: Patients 16 years old or younger at the time of injury, admitted to PICU for management of TBI with TBI-related pathology confirmed on CT or MRI and clinical requirement for invasive monitoring of ICP and ABP, will be eligible for recruitment. There are no exclusion criteria for the project.

Patient Recruitment and Consent: Each PTBI patient admitted to the recruiting PICU with invasive monitoring of ICP and ABP is considered for recruitment. The research team at each recruiting PICU identifies the patients and sets up high-resolution data collection to the project laptop under an anonymised ID as soon as possible after the patient has been commenced on invasive monitoring and stabilized on PICU. Physiological and clinical data is recorded under this unique study ID. As the acute phase of the project is purely observational and does not involve any therapeutic intervention or change in regular standard care, we take a deferred consent for acute data collection and sharing. This is important to obtain a bias-free sample and ensure capture of early changes in intracranial physiology after head injury. When appropriate, the identified guardian/parent is informed and consented for data sharing and follow-up before the patient is discharged from the hospital. For patients who die before the consent can be obtained, the clinician in charge of patient's clinical care only (not study team) will access and anonymise data of the deceased patient under his/her clinical care; this anonymised data will then be provided to the study team.

Data Collection: ICP will be monitored with an intraparenchymal microsensor inserted into the right (predominately but can be left) frontal cortex and ABP will be monitored in the radial or femoral artery with a zero calibration at the level of the right atrium. The head end elevation is part of the standard international protocol for management for PTBI, and any deviations from this policy in individual cases will be recorded.

For peer review only - http://bmjopen.bmj.com/site/about/guidelines.xhtml

Full resolution data of routinely monitored physiological parameters in managing PTBI [ICP, ABP, $ETCO_2$, SpO_2 and ECG] will be collected from bed-side monitors. Pulsatile signals [ICP, ABP and ECG] will be recorded with the maximum sampling frequency offered by the bed-side monitors, at least 100 Hz for invasive pressures, and at least 200Hz for ECG. This will be done using relevant, monitor type specific, data export protocols implemented in the study (ICM+) software running on a bed-side laptop. The same software will be later used for the retrospective analysis of all stored signals. The continuous data collection will be stopped when the patient either starts to wake up or dies and the invasive monitoring is stopped. Apart from general demographics and clinical data, the other data being collected are mechanism and details of injury, relevant past history, post resuscitation Glasgow coma scale, initial pupillary size and reactivity, pre-hospital hypoxia/ hypotension/ hyperthermia/ cardiopulmonary resuscitation, injury & disease severity scores (Pediatric Index of Mortality- PIM II score, Injury Severity Score- ISS), CT scan findings. We are also collecting: daily laboratory values (hemoglobin, glucose, sodium, lactate), PaO_2, $PaCO_2$ and FiO_2, PEEP, medical interventions for TBI (positioning, collar, temperature, CO_2, glucose, hypertonic saline or mannitol, sedation/paralysis, barbiturate coma, anti-seizure medication, use of vasoactive drugs to maintain blood pressure & surgical interventions (haematoma evacuation, elevation of depressed fracture, CSF diversion-external ventricular drainage, new brain imaging, any other relevant event.

<u>Data Management and Monitoring</u>: Each patient is allocated a unique study ID and all the collected data is anonymised to this ID. Appointed data manager is responsible for monitoring data transfer to Cambridge, for data verification, validation and repackaging, and storing it in the archive location on the server. Full ethical and legal practice is being followed and database will be managed securely under the supervision of data custodian. The project is funded for five years (30th June 2023) at present and we will endeavor to get further funding to carry on the data collection and database further in the near future. The data will be stored on a secure server located on a private network behind a tight firewall at the sponsoring institute. We will use the already established system in use for similar larger adult project, CENTER-TBI (https://www.center-tbi.eu/). Briefly, all the physiological time series data collected in each participating site using ICM+ software are stripped of any identifiers and packaged into the HDF5 (https://www.hdfgroup.org/) based format. The file is then uploaded using the SSH data transfer protocol function of ICM+ to the Cambridge server using public/private key authentication and the username/password distributed in an encrypted format and installed on the ICM+ laptop. The data comes into ‘upload’ folder on that server and is then promptly moved to a staging folder, inaccessible

For peer review only - http://bmjopen.bmj.com/site/about/guidelines.xhtml

via the SSH connection. The data is then scrutinized for potential problems, artifacts, stripped of any potential identifiers embedded into the annotations, stripped of the data recording date (the timestamps are recorded as relative to the date of ictus, not kept with the data file) and archived permanently. The original data is then removed. All analysis is subsequently done on that pre-processed data. The data is backed up daily, using incremental backup procedure.

Outcome Measures: The primary outcome will be measured by Glasgow Outcome Scale Extended Pediatric version (GOSE-Peds) at 12-months post-injury against the PRx.

The study will also include the following secondary outcome measures:

- GOSE-Peds at 6 months against the PRx
- Delta CPP (CPP deviation from CPPopt) against the GOSE-Peds at 6 & 12 months
- Rate of severe TBI-related surgical interventions undertaken during the index admission
- Mortality (30-day, 6 months, 12 months)
- Length of stay in hospital and rehabilitation

There is more detailed neuropsychology assessment at 12 months follow-up by using telephone-based interviews and postal questionnaires with parents by using the following tests (child versions will be used where available): BRIEF, PedsQL, SDQ, CCC, CASP and RCADS. In addition, with parental permission, school teacher will be contacted for SDQ and BRIEF with postal questionnaires.

Due to the covid-related impact on research, to circumvent the missed follow-ups for the recruited patients:

1. Where possible, site teams will carry out telephone interview with parents/legal guardian to ascertain child's neuro-developmental performance at the 1st anniversary of child's head injury for retrospective GOSE-Peds score at 12 months. There is precedence to this approach, especially in children where the developmental milestones/neurodevelopment are judged from memory based on how an individual child performed at different time-points in their developmental life (17-20).

2. If the child was seen in Acquired Brain Injury/Neurology/Neuro-psychology/Neurodevelopmental clinic at a specific site, the local research team will review the clinic letters/notes to ascertain child's outcome as close as possible to 12 months from the head injury.

For peer review only - http://bmjopen.bmj.com/site/about/guidelines.xhtml

Statistics

Power Calculation: The sample size has been calculated assuming a mean (± SD) PRx of 0.03 ± 0.13 in patients with favourable outcome and a mean (± SD) PRx of 0.10 ± 0.17 (SD) for patients with unfavourable outcome (from our previous work) (6). For a one-sided analysis, using a favourable/unfavourable ratio of 0.77, with 80% power and alpha error of 5%, and allowing for losses (protocol violations, withdrawal of consent, or loss of follow up) the study will require 135 patients. Recruiting 135 patients with severe TBI in 36 months should have been feasible as based on the national audits of PICU [PICAnet report 2015: 188, 210, 211 patients in 2013, 2014 and 2015 respectively with ICP devices from the 10 UK centres recruiting into the current study] and major trauma [TARN report on 2 years of severe injury in children (2013-2014): 1018 children in two years with severe head injury] with an estimated recruitment rate of 5-7 patients per site per year. On the basis of hospital episode statistics (HES) and data from the on-going local severe traumatic brain injury audit, approximately 5-10 patients with severe TBI are admitted in a medium-sized PICU each year.

However, due to the Covid-19 pandemic, the recruitment has been slow (pause of non-covid research projects, research staff re-deployment to clinical work and lockdowns leading to changes in trauma patterns in children), and the study has been extended until December 2024 in agreement with the sponsor and funding organisation.

Statistical Analysis: Continuous variables will be assessed for normality and expressed as mean (SD) or median (interquartile range), depending on the underlying distribution of the data. Categorical variables will be reported as counts and proportions. Differences in physiological values between survivors and non-survivors will be interrogated with the Mann-Whitney U-test for non-normally distributed continuous variables, Student t-test for normally distributed continuous variables and Fisher exact test for categorical variables. All tests will be two-tailed and unadjusted for multiple comparisons. Spearman rank correlation coefficient (ρ) will be used to see correlations between variables. Identification of critical threshold values of PRx for predicting unfavorable outcome will be performed using iterative chi-square tests, as per the method described by Sorrentino et al (21). To account for any possible confounding effect of the differing periods of monitoring and day post admission on which monitoring starts on outcome associations, ordinal logistic regression analysis will be performed. The independent effect of each continuously monitored variable on GOSE-Peds will be assessed by including the length of monitoring and monitoring commencement day post admission as covariates in the regression models. Where appropriate PRx will be Fisher transformed to remove the <-1;1> constrain and ensure normalization of its distribution. The effect of time on PRx will be assessed using repeated-measures analysis of variance with each patient treated as a random effect.

For peer review only - http://bmjopen.bmj.com/site/about/guidelines.xhtml

Ethics Approval & Considerations: Ethical approval was provided by Health Research Authority, South West- Central Bristol Research Ethics Committee (Ref: 18/SW/0053). As the project has approval for research database, it did not require site specific ethics. The project was reviewed and approved at each participating site before starting recruitment. The study protocol, patient information sheet, consent forms, and other study related documents were reviewed and approved by the REC with regards to their scientific content and compliance with applicable research regulations involving human subjects. The current study protocol is v5 dated 28/08/2018.

Participant Confidentiality and data protection: The data collection part of this research study is limited to accessing, collecting and analyzing information that is monitored and placed in the medical record as part of the child's standard care while being treated for severe TBI in PICU. Our plan to protect patient-subject identifiers from improper use and disclosure is consistent with our plans to protect the overall data of the study. Specifically, access to patient-subject identifiers will be limited to study personnel that require its use for clinical reasons. The anonymised data will be used by the investigators and research staff listed on this application which will help ensure that there is no improper use or disclosure. Secondly, a limited amount of personal information (specifically, only the month and year of birth which is essential to establishing the age of the subject) will be collected. Third, all patient-subject identifiers will be kept in a locked cabinet within the recruiting hospital, which can only be accessed by the study staff on the delegation log. Fourth, all subjects within the study will be given a unique, de-identified study ID and this study ID will be utilized for importing data to the study database. We believe that our plan adequately ensures that patient-subject identifiers will be protected from improper use and disclosure. There will be no patient identifiable information shared outside the respective clinical teams looking after each patient. Within each individual centre, the patient identifiable information will not be seen/shared with anyone who does not access it otherwise (outside the clinical teams). Within the centres, the consent forms and the data collection will be stored in a secure locked cupboard within a secure locked room. The individual centres will be instructed to save the data for five years (to ensure data auditing and clearance), after which time the collected identifiable data will be destroyed).

The project is to be carried out in conformation with the spirit and the letter of the declaration of Helsinki, and in accord with the ICH Good Clinical Practice Guidelines. The application, the database management and monitoring have full support from the patient safety department at sponsoring institute, and the protocol has been approved by them. The entire project will be conducted under close scrutiny of data custodian and the patient safety department.

For peer review only

For peer review only - http://bmjopen.bmj.com/site/about/guidelines.xhtml

Dissemination: The results will be published in peer-reviewed journals and presented at relevant national and international conferences and seminars.

Patient and Public Involvement: We work closely with the Children's Brain Injury Trust and have a child and family worker embedded in the team; they have given their input from patients and public perspective into the study protocol & design which has been acceptable to patients and families.

The patients are not being directly consented for the study as the children and young people who are eligible for the study are not able to give meaningful informed consent or assent from the very nature of the disease making them eligible for inclusion to the study. The data is only shared and stored after consent from the patient's family or legal guardians; the consent is also sought for the follow-up. Although we will not be able to tell the results to individual patients, the results will be published in peer-reviewed journals. Since this is not an interventional study, there is no research burden to an individual patient.

Conclusion: The project will provide vital information on thresholds of PRx and trends of optimal CPP in children to limit secondary brain injury. It will also pave way to further scientific research by establishing a database for this complex but infrequent disease in children.

For peer review only

For peer review only - http://bmjopen.bmj.com/site/about/guidelines.xhtml

Acknowledgements: The authors thank Ricardo G Branco, Joseph Donnelly and Matthew Garnett for their valuable contribution to the study design. The authors also thank all the research and clinical staff at the participating sites: Birmingham Children's Hospital, Great Ormond Street Hospital, Leeds General Hospital, Manchester Children's Hospital, Queen's Medical Centre Nottingham, John Radcliffe Hospital Oxford, Royal London Hospital, Sheffield Children's Hospital and Southampton Children's Hospital.

Contributors: SA is the chief investigator, DW and ED are study nurses, MP and MC have been data coordinators and research associates for the study providing technical support, SW has contributed to neuro-developmental testing. PS, MC, AY, AM and PH are trial co-applicants. All the authors have read and approved the final version of this manuscript. SA, PS, MC, PH are part of the study steering committee.

Funding: This study is funded by Action Medical Research for Children's Charity, UK. Cambridge University Hospitals is the study sponsor (contact person: Michelle Ellerbeck- michelle.ellerbeck@nhs.net).

Conflict of interest: ICM+ is a software licensed by Cambridge Enterprise Ltd. PS and MC have a financial interest in a part of licensing fee; the licensing fee was waivered for this study. There are no additional patents, products in development or marketed products to declare.

Data Access: The final database will be saved on a non-public secure repository, the access to the data will be available on request.

For peer review only

References:

1. Faul M, Xu L, Wald MM, Coronado VG. Traumatic brain injury in the United States: emergency department visits, hospitalizations, and deaths. Atlanta (GA): Centers for Disease Control and Prevention, National Center for Injury Prevention and Control; 2010.

2. Dewan MC MN, Wellons III C, Bonfield CM. Epidemiology of Global Pediatric Traumatic Brain Injury: Qualitative Review. World Neurosurg 2016;91:497-509.

3. Langlois JA, Rutland-Brown W, Thomas KE. Traumatic Brain Injury in the United States: Emergency Department Visits, Hospitalizations, and Deaths. Atlanta, GA: Centers for Disease Control and Prevention; 2004.

4. Bayreuther J, Wagener S, Woodford M, et al. Paediatric trauma: Injury pattern and mortality in the UK. Arch Dis Child Educ Pract Ed 2009;94:37-41

5. Kochanek PM, Tasker RC, Nancy C, et al,. Guidelines for the management of pediatric severe traumatic brain, third edition: update of the Brain Trauma Foundation Guidelines. Pediatr Crit Care Med. 2019;20:S1-82.

6. Young AM, Donnelly J, Czosnyka M, et al. Continuous multimodality in children after traumatic brain injury-preliminary experience. PLoS One 2016;11:e0148817. doi: 10.1371/journal.pone.0148817.

7. Lewis PM, Czosnyka M, Carter BG, et al. Cerebrovascular pressure reactivity in children with traumatic brain injury. Pediatr Crit Care Med 2015;16:739-49.

8. Brady KM, Shaffner DH, Lee JK, et al. Continuous monitoring of cerebrovascular pressure reactivity after traumatic brain injury in children. Pediatrics 2009;124:e1205-12. doi: 10.1542/peds.2009-0550.

9. Aries MJ, Czosnyka M, Budohoski KP, et al, Continuous determination of optimal cerebral perfusion pressure in traumatic brain injury. Crit Care Med. 2012;40:2456-63.

10. Steiner LA, Czosnyka M, Piechnik SK, et al. Continuous monitoring of cerebrovascular pressure reactivity allows determination of optimal cerebral perfusion pressure in patients with traumatic brain injury. Crit Care Med. 2002;30:733–8.

11. Lang EW, Chesnut RM: Intracranial pressure and cerebral perfusion pressure in severe head injury. New Horiz 1995; 3:400–409.

12. Paulson OB, Strandgaard S, Edvinsson L: Cerebral autoregulation. Cerebrovasc Brain Metab Rev 1990; 2:161–192.

13. Le Roux P, Menon DK, Citerio G, et al,. The International Multidisciplinary Consensus Conference on Multimodality Monitoring in Neurocritical Care: Evidentiary Tables: A Statement for Healthcare Professionals from the Neurocritical Care Society and the European Society of Intensive Care Medicine. Neurocrit Care. 2014;21:S297-361.

14. Czosnyka M, Smielewski P, Kirkpatrick P, Laing RJ, Menon D, Pickard JD. Continuous assessment of the cerebral vasomotor reactivity in head injury. Neurosurgery. 1997;41:11–7.

15. Johnson U, Lewen A, Ronne-Engstrom E, et al. Should the neuro-intensive care management of traumatic brain injury patients be individualized according to autoregulation status and injury subtype? Neurocrit Care 2014;21:259-65.

16. Jaeger M, Lang EW. Cerebrovascular pressure reactivity and cerebral oxygen regulation after severe head injury. Neurocrit Care 2013;19:69-73.

17. Bejerot S, Lugnegard T. Retrospective parental assessment of childhood neurodevelopmental problems: the use of the Five to Fifteen questionnaire in adults. B J Psych Open 2019;5:1-5. doi:10.1192/bjo.2019.30.

For peer review only - http://bmjopen.bmj.com/site/about/guidelines.xhtml

18. Lord C, Rutter M, Couteur AL. Autism diagnostic interview- revised: A revised version of a diagnostic interview for caregivers of individuals with possible pervasive developmental disorders. J Autism Developmental Disorders 1994;24:659-685.

19. Fombonne E. Diagnostic assessment in a sample of autistic and developmentally impaired adolescents. J Autism Developmental Disorders 1992;22:563-581.

20. Hirvikoski T, Jokinen SLJ, Renhorn E, Trillingsgaard A, Kadesjo B, Borg J, The FTF working group. Using the five to fifteen collateral informant questionnaire for retrospective assessment of childhood symptoms in adults with and without autism or ADHD. Eur Child Adol Psych 2021;30:1367-1381.

21. Sorrentino E, Diedler J, Kasprowicz M, Budohoski KP, Haubrich C, Smielewski P, Outtrim JG, Manktelow A, Hutchinson PJ, Pickard JD, Menon DK, Czosnyka M. Critical thresholds for cerebrovascular reactivity after traumatic brain injury. Neurocrit Care 2012;16:258-66

For peer review only

For peer review only - http://bmjopen.bmj.com/site/about/guidelines.xhtml

Figure 1:

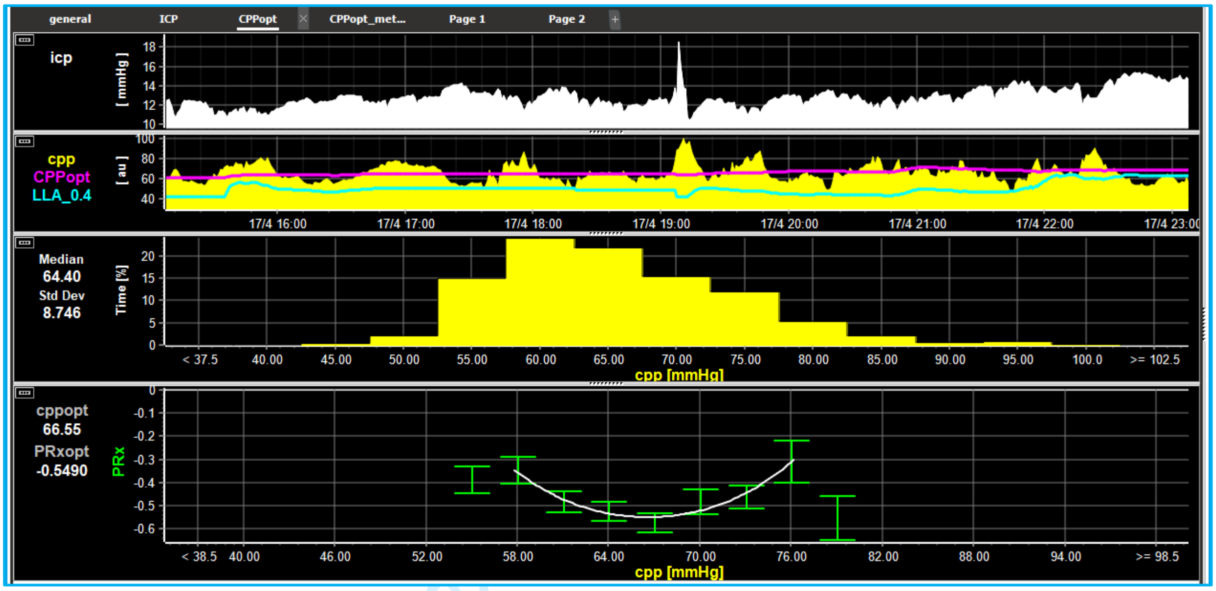
general
ICP
CPPopt
CPPopt_met...
Page 1
Page 2
icp
[mmHg]
cpp
CPPopt
LLA_0.4
[au]
17/4 16:00
17/4 17:00
17/4 18:00
17/4 19:00
17/4 20:00
17/4 21:00
17/4 22:00
Median
64.40
Std Dev
8.746
Time [%]
cpp [mmHg]
cppopt
66.55
PRxopt
-0.5490
PRx
cpp [mmHg]

Reporting checklist for protocol of a clinical trial.

Based on the SPIRIT guidelines.

Instructions to authors

Complete this checklist by entering the page numbers from your manuscript where readers will find each of the items listed below.

Your article may not currently address all the items on the checklist. Please modify your text to include the missing information. If you are certain that an item does not apply, please write "n/a" and provide a short explanation.

Upload your completed checklist as an extra file when you submit to a journal.

In your methods section, say that you used the SPIRITreporting guidelines, and cite them as:

Chan A-W, Tetzlaff JM, Gøtzsche PC, Altman DG, Mann H, Berlin J, Dickersin K, Hróbjartsson A, Schulz KF, Parulekar WR, Krleža-Jerić K, Laupacis A, Moher D. SPIRIT 2013 Explanation and Elaboration: Guidance for protocols of clinical trials. BMJ. 2013;346:e7586

For peer review only

		Reporting Item	Page Number
**Administrative information**			
Title	#1	Descriptive title identifying the study design, population, interventions, and, if applicable, trial acronym	1
Trial registration	#2a	Trial identifier and registry name. If not yet registered, name of intended registry	1
Trial registration: data set	#2b	All items from the World Health Organization Trial Registration Data Set	
Protocol version	#3	Date and version identifier	9
Funding	#4	Sources and types of financial, material, and other support	11
Roles and responsibilities: contributorship	#5a	Names, affiliations, and roles of protocol contributors	1, 11

Roles and responsibilities: sponsor contact information	#5b	Name and contact information for the trial sponsor	11
Roles and responsibilities: sponsor and funder	#5c	Role of study sponsor and funders, if any, in study design; collection, management, analysis, and interpretation of data; writing of the report; and the decision to submit the report for publication, including whether they will have ultimate authority over any of these activities	11
Roles and responsibilities: committees	#5d	Composition, roles, and responsibilities of the coordinating centre, steering committee, endpoint adjudication committee, data management team, and other individuals or groups overseeing the trial, if applicable (see Item 21a for data monitoring committee)	11
**Introduction**			
Background and rationale	#6a	Description of research question and justification for undertaking the trial, including summary of relevant studies (published and unpublished) examining benefits and harms for each intervention	3
Background and rationale: choice of comparators	#6b	Explanation for choice of comparators	3
Objectives	#7	Specific objectives or hypotheses	4
Trial design	#8	Description of trial design including type of trial (eg, parallel group, crossover, factorial, single group), allocation ratio, and framework (eg, superiority, equivalence, non-inferiority, exploratory)	4-7
**Methods: Participants, interventions, and outcomes**			
Study setting	#9	Description of study settings (eg, community clinic, academic hospital) and list of countries where data will be collected. Reference to where list of study sites can be obtained	5

For peer review only

Eligibility criteria	#10	Inclusion and exclusion criteria for participants. If applicable, eligibility criteria for study centres and individuals who will perform the interventions (eg, surgeons, psychotherapists)	5
Interventions: description	#11a	Interventions for each group with sufficient detail to allow replication, including how and when they will be administered	5-6
Interventions: modifications	#11b	Criteria for discontinuing or modifying allocated interventions for a given trial participant (eg, drug dose change in response to harms, participant request, or improving / worsening disease)	NA
Interventions: adherance	#11c	Strategies to improve adherence to intervention protocols, and any procedures for monitoring adherence (eg, drug tablet return; laboratory tests)	NA
Interventions: concomitant care	#11d	Relevant concomitant care and interventions that are permitted or prohibited during the trial	NA
Outcomes	#12	Primary, secondary, and other outcomes, including the specific measurement variable (eg, systolic blood pressure), analysis metric (eg, change from baseline, final value, time to event), method of aggregation (eg, median, proportion), and time point for each outcome. Explanation of the clinical relevance of chosen efficacy and harm outcomes is strongly recommended	7
Participant timeline	#13	Time schedule of enrolment, interventions (including any run-ins and washouts), assessments, and visits for participants. A schematic diagram is highly recommended (see Figure)	NA
Sample size	#14	Estimated number of participants needed to achieve study objectives and how it was determined, including clinical and statistical assumptions supporting any sample size calculations	8
Recruitment	#15	Strategies for achieving adequate participant enrolment to reach target sample size	5
**Methods: Assignment of interventions (for controlled trials)**			
Allocation: sequence generation	#16a	Method of generating the allocation sequence (eg, computer-generated random numbers), and list of any factors for stratification. To reduce predictability of a random sequence, details of any planned restriction (eg, blocking) should be	NA

For peer review only

		provided in a separate document that is unavailable to those who enrol participants or assign interventions	
Allocation concealment mechanism	#16b	Mechanism of implementing the allocation sequence (eg, central telephone; sequentially numbered, opaque, sealed envelopes), describing any steps to conceal the sequence until interventions are assigned	NA
Allocation: implementation	#16c	Who will generate the allocation sequence, who will enrol participants, and who will assign participants to interventions	NA
Blinding (masking)	#17a	Who will be blinded after assignment to interventions (eg, trial participants, care providers, outcome assessors, data analysts), and how	NA
Blinding (masking): emergency unblinding	#17b	If blinded, circumstances under which unblinding is permissible, and procedure for revealing a participant's allocated intervention during the trial	NA
**Methods: Data collection, management, and analysis**			
Data collection plan	#18a	Plans for assessment and collection of outcome, baseline, and other trial data, including any related processes to promote data quality (eg, duplicate measurements, training of assessors) and a description of study instruments (eg, questionnaires, laboratory tests) along with their reliability and validity, if known. Reference to where data collection forms can be found, if not in the protocol	5-7
Data collection plan: retention	#18b	Plans to promote participant retention and complete follow-up, including list of any outcome data to be collected for participants who discontinue or deviate from intervention protocols	5-7
Data management	#19	Plans for data entry, coding, security, and storage, including any related processes to promote data quality (eg, double data entry; range checks for data values). Reference to where details of data management procedures can be found, if not in the protocol	6-7
Statistics: outcomes	#20a	Statistical methods for analysing primary and secondary outcomes. Reference to where other details of the statistical analysis plan can be found, if not in the protocol	7-8

For peer review only

Statistics: additional analyses	#20b	Methods for any additional analyses (eg, subgroup and adjusted analyses)	8
Statistics: analysis population and missing data	#20c	Definition of analysis population relating to protocol non-adherence (eg, as randomised analysis), and any statistical methods to handle missing data (eg, multiple imputation)	NA
**Methods: Monitoring**			
Data monitoring: formal committee	#21a	Composition of data monitoring committee (DMC); summary of its role and reporting structure; statement of whether it is independent from the sponsor and competing interests; and reference to where further details about its charter can be found, if not in the protocol. Alternatively, an explanation of why a DMC is not needed	11
Data monitoring: interim analysis	#21b	Description of any interim analyses and stopping guidelines, including who will have access to these interim results and make the final decision to terminate the trial	NA
Harms	#22	Plans for collecting, assessing, reporting, and managing solicited and spontaneously reported adverse events and other unintended effects of trial interventions or trial conduct	NA
Auditing	#23	Frequency and procedures for auditing trial conduct, if any, and whether the process will be independent from investigators and the sponsor	NA
**Ethics and dissemination**			
Research ethics approval	#24	Plans for seeking research ethics committee / institutional review board (REC / IRB) approval	9
Protocol amendments	#25	Plans for communicating important protocol modifications (eg, changes to eligibility criteria, outcomes, analyses) to relevant parties (eg, investigators, REC / IRBs, trial participants, trial registries, journals, regulators)	7
Consent or assent	#26a	Who will obtain informed consent or assent from potential trial participants or authorised surrogates, and how (see Item 32)	5

For peer review only

Consent or assent: ancillary studies	#26b	Additional consent provisions for collection and use of participant data and biological specimens in ancillary studies, if applicable	NA
Confidentiality	#27	How personal information about potential and enrolled participants will be collected, shared, and maintained in order to protect confidentiality before, during, and after the trial	9
Declaration of interests	#28	Financial and other competing interests for principal investigators for the overall trial and each study site	11
Data access	#29	Statement of who will have access to the final trial dataset, and disclosure of contractual agreements that limit such access for investigators	11
Ancillary and post trial care	#30	Provisions, if any, for ancillary and post-trial care, and for compensation to those who suffer harm from trial participation	
Dissemination policy: trial results	#31a	Plans for investigators and sponsor to communicate trial results to participants, healthcare professionals, the public, and other relevant groups (eg, via publication, reporting in results databases, or other data sharing arrangements), including any publication restrictions	10
Dissemination policy: authorship	#31b	Authorship eligibility guidelines and any intended use of professional writers	
Dissemination policy: reproducible research	#31c	Plans, if any, for granting public access to the full protocol, participant-level dataset, and statistical code	
**Appendices**			
Informed consent materials	#32	Model consent form and other related documentation given to participants and authorised surrogates	
Biological specimens	#33	Plans for collection, laboratory evaluation, and storage of biological specimens for genetic or molecular analysis in the current trial and for future use in ancillary studies, if applicable	

None The SPIRIT Explanation and Elaboration paper is distributed under the terms of the Creative Commons Attribution License CC-BY-NC. This checklist can be completed online using https://www.goodreports.org/, a tool made by the EQUATOR Network in collaboration with Penelope.ai

For peer review only

BMJ Open

Studying Trends of Auto-Regulation in Severe Head Injury in Paediatrics (STARSHIP): Protocol to study cerebral autoregulation in a prospective multicentre observational research database study.

Journal:	*BMJ Open*
Manuscript ID	bmjopen-2023-071800.R1
Article Type:	Protocol
Date Submitted by the Author:	17-Feb-2023
Complete List of Authors:	Agrawal, Shruti; Cambridge University Hospitals NHS Foundation Trust, Paediatric Intensive Care; Cambridge University Hospitals<br>Placek, Michal M.; University of Cambridge, Department of Clinical Neurosciences<br>White, Deborah; Cambridge University Hospitals NHS Foundation Trust, Paediatric Intensive Care<br>Daubney, Esther; Cambridge University Hospitals NHS Foundation Trust, Paediatric Intensive Care<br>Cabeleira, Manuel; University College London<br>Smielewski, Peter; University of Cambridge, Clinical Neuroscience<br>Czosnyka, Marek; University of Cambridge,<br>Young, Adam; University of Cambridge, Department of Clinical Neurosciences<br>Watson, Suzanna; Cambridge Centre for Paediatric Neuropsychological Rehabilitation, Paediatric Neuropsychology; Cambridge University Hospitals NHS Foundation Trust, Centre for Child Development<br>Maw, Anna; Cambridge University Hospitals NHS Foundation Trust, Paediatric Neurologi<br>Hutchinson, Peter; University of Cambridge, Academic Neurosurgery
**Primary Subject Heading**:	Paediatrics
Secondary Subject Heading:	Intensive care
Keywords:	Paediatric intensive & critical care < PAEDIATRICS, Physiology < NATURAL SCIENCE DISCIPLINES, Neurological injury < NEUROLOGY, TRAUMA MANAGEMENT

SCHOLARONE™
Manuscripts

Studying Trends of Auto-Regulation in Severe Head Injury in Paediatrics (STARSHIP): Protocol to study cerebral autoregulation in a prospective multicentre observational research database study.

Shruti Agrawal MD[1,2], Michal Placek PhD[3], Deborah White MSc[1], Esther Daubney BSc[1], Manuel Cabeleira MSc[4], Peter Smielewski PhD[3], Marek Czosnyka PhD[3,5], Adam Young PhD[3], Suzanna Watson PhD[6,7], Anna Maw FRCPCH[7], Peter J Hutchinson PhD, FMedSci[3]

1. Paediatric Intensive Care, Cambridge University Hospitals, Cambridge, UK
2. Department of Paediatrics, University of Cambridge, UK
3. Division of Neurosurgery, Department of Academic Neurosciences, University of Cambridge UK
4. Department of Mechanical Engineering, University College London, UK
5. ISE, Warsaw University of Technology, Poland
6. Cambridge Centre for Paediatric Neuropsychological Rehabilitation, Cambridge, UK
7. Centre for Child Development, Paediatric Neurology, Cambridge University Hospitals, Cambridge, UK

Corresponding Author:

Dr Shruti Agrawal

PICU, Level 3, Box 7

Addenbrookes Hospital

Hills Road, Cambridge CB2 0QQ

Email: shruti.agrawal1@nhs.net, sa882@cam.ac.uk

Ph: +441223348066

Word Count: 3441

Trial Registration: NCT05688462

For peer review only

For peer review only - http://bmjopen.bmj.com/site/about/guidelines.xhtml

Abstract:

Introduction: Studying cerebral autoregulation, particularly PRx (pressure reactivity index), is commonly employed in adult traumatic brain injury (TBI) and gives real-time information about intracranial pathophysiology which can help in patient management. Experience in paediatric TBI (PTBI) is limited to single centre studies despite disproportionately higher incidence of morbidity and mortality in PTBI than in adult TBI.

Project: We describe the protocol to study cerebral autoregulation using PRx in PTBI. The project called STARSHIP (**St**udying **T**rends of **A**uto-**R**egulation in **S**evere **H**ead **I**njury in **P**aediatrics) is a multi-centre prospective ethics approved research database study from 10 centres across the UK. Recruitment started in July 2018 with financial support from local/national charities (Action Medical Research for Children, UK).

Methods and Analysis: The first phase of the project is powered to detect optimal thresholds of PRx associated with favourable outcome in PTBI by recruiting 135 patients (initial target of 3 years which has changed to 5 years due to delays related to Covid-19 pandemic) from 10 centres in the UK with outcome follow-up to one year post ictus. The secondary objectives are to characterize patterns of optimal cerebral perfusion (CPPopt) in PTBI and compare the fluctuations in these measured parameters with outcome. The goal is to create a comprehensive research database of a basic set of high-resolution (full waveforms resolution) neuro-monitoring data in PTBI for scientific use.

Ethics and Dissemination: Favourable ethical approval has been provided by Health Research Authority, Southwest-Central Bristol Research Ethics Committee (Ref: 18/SW/0053). Results will be disseminated via publications in peer reviewed medical journals and presentations at national and international conferences.

Key words: Paediatric Traumatic Brain Injury, children, cerebral autoregulation, pressure reactivity index (PRx), optimal cerebral perfusion pressure.

Strengths and Limitations:

- STARSHIP is the first prospective multi-centre cohort study in children powered to look at the association of cerebral autoregulation as studied by PRx with the neuro-developmental outcome after severe TBI.
- It will create a comprehensive research database of high-resolution neuro-monitoring data with neurodevelopmental follow-up in PTBI for scientific use.
- It will characterize patterns of optimal cerebral perfusion (CPPopt) in PTBI and compare the

For peer review only - http://bmjopen.bmj.com/site/about/guidelines.xhtml

For peer review only

fluctuations in these measured parameters with outcome.

For peer review only

For peer review only - http://bmjopen.bmj.com/site/about/guidelines.xhtml

Introduction: Traumatic brain injuries (TBI) are a major burden to public health with a high incidence and potential long term consequences (1). Incidence of TBI in children ranges from 47 to 280 per 100,000 and are a leading cause of hospital admission and death each year (2). Epidemiological studies have demonstrated that the incidence of hospitalisation, fatal brain injury, and morbidity is disproportionately higher in children (3,4). Despite this, little is known about specific aspects of the paediatric TBI (PTBI) pathology, with most of the knowledge extrapolated from adults. Following head trauma, primary brain injury occurs as a result of the initial trauma. This triggers a cascade of secondary insults in hours to days following primary injury (for e.g., insufficient blood flow and oxygen supply, low blood pressure, brain swelling, increased intracranial pressure, etc.), resulting in secondary brain injury. The secondary brain injury plays significant role in outcome. Therefore, the focus of standard management of PTBI in Paediatric Intensive Care Units (PICU) is to limit these secondary brain insults (5). Current evidence and guidelines suggest use of intracranial pressure (ICP) and cerebral perfusion pressure [CPP; defined as arterial blood pressure (ABP) minus ICP] measurements to guide therapies (5). Although there is some consensus on what level of ICP is unacceptably high, the optimal level of CPP is controversial in children. We and others have used advanced monitoring to investigate the association between CPP and clinical outcome (6-8) and examined means of establishing individualised targets for CPP management, termed 'CPP Optimal' (CPPopt), based on analysis of the status of cerebral vascular reactivity, interpreted as a surrogate measure of autoregulation of cerebral blood flow (CBF). Interestingly, CPPopt shows significant variation from the guidelines recommendation, and CPPopt can be different between patients and change over time in each patient (9,10). This phenomenon is due to dysfunction and variability of autoregulatory mechanisms in patients with severe TBI that control cerebral blood flow (and CPPopt) (11, 12). In adults and in our preliminary study in children, we found that the difference between CPP recommended by the guidelines and CPPopt was associated with worsened clinical outcome (6,9,10). For this reason, indices of cerebral autoregulation have been recommended to guide management of severe TBI in adults (13), but so far there is limited evidence to support their routine use in children (7,8).

Normally, all severe PTBI in PICUs have continuous ICP and ABP monitoring; these values can be used to determine state of cerebrovascular autoregulation and subsequently CPPopt. In health, fluctuations in systemic circulation are compensated by reactive changes in cerebral vasomotor tone to maintain steady cerebral blood flow. For example, a drop in ABP induces vasodilatation, which increases cerebral blood volume and ICP. Conversely, an increase in ABP leads to vasoconstriction, which decreases cerebral blood volume and ICP. This relationship can be studied continuously in real time through the correlation of fluctuations in ABP and ICP

For peer review only - http://bmjopen.bmj.com/site/about/guidelines.xhtml

waveforms as the cerebrovascular pressure reactivity index (PRx) (14). This index can range from −1 to +1, with negative or zero values indicating intact pressure reactivity and normal autoregulation (associated with better clinical outcome), while positive values indicating disturbed pressure reactivity and abnormal autoregulation (associated with poorer outcome) (12). PRx has been found useful prognostic marker in adult TBI patients (14-16). Plotting PRx against measured CPP can determine the CPP at which the vasculature is most reactive, therefore estimating CPPopt for an individual patient at a particular point in time (Figure 1). To date, apart from our experience (6), only two other small paediatric data sets have been published about PRx and CPPopt (7,8). This study aims to provide vital information on optimal thresholds of PRx and CPP in children to limit secondary brain injury and improve outcomes.

Study Aim: We aim to create a research database of high-resolution monitoring data from PTBI patients along with the clinical and demographic details and neuro-psychological outcome up to 12 months post-ictus, from 10 UK PICUs to be used for research purposes. As the first step, we will analyze the data to determine retrospective optimal clinical parameters when managing these patients. Our hypothesis is that we can define the optimal parameters for CPP based on indices of autoregulation (PRx) and that the optimal parameters of CPP and PRx in PTBI will differ to adults, both qualitatively and quantitatively.

Study Objectives:

Primary Objective: To detect thresholds of PRx associated with favourable outcome in PTBI.

Secondary objectives:

1. Characterise patterns of optimal CPP in PTBI as a function of age, severity and character of injury, intracranial pressure, etc.
2. Compare the fluctuations in these measured parameters with the outcome.
3. Establish a comprehensive paediatric database of a basic set of high resolution (pulse waveforms resolution) neuro-monitoring data including ICP, ABP and ECG.

Methods and Analysis: This is an observational prospective cohort study. Children admitted to ten identified PICUs in the UK with severe TBI with a clinical need for ICP and ABP monitoring will be included for data collection. The database will contain routinely monitored physiological parameters during management of PTBI in PICU. Without disturbing the routine care and management of these patients, we aim to collect this data

For peer review only - http://bmjopen.bmj.com/site/about/guidelines.xhtml

continuously in a bedside laptop equipped with the study software (ICM+, Cambridge Enterprise Ltd, Cambridge, UK). Alongside these physiological parameters, we will also collect relevant clinical and demographic data (details below) and follow these patients for up to 12 months post ictus to assess neuro-psychological outcome. The created database will be used to analyze the state of cerebral autoregulation. We will also analyze the data to see if we can derive optimal cerebral perfusion pressures based on the state of autoregulation and compare it with the patient outcome at 6 and 12 months. The database can also be subsequently used in the future projects testing hypotheses inspired by the experience gained in the adult TBI population research & to answer important questions about severe PTBI.

Patient and Public Involvement: We work closely with the Children's Brain Injury Trust and have a child and family worker embedded in the team; they have given their input from patients and public perspective into the study protocol & design which has been acceptable to patients and families.

The patients are not being directly consented for the study as the children and young people who are eligible for the study are not able to give meaningful informed consent or assent from the very nature of the disease making them eligible for inclusion to the study. The data is only shared and stored after consent from the patient's family or legal guardians; the consent is also sought for the follow-up. Although we will not be able to tell the results to individual patients, the results will be published in peer-reviewed journals. Since this is not an interventional study, there is no research burden to an individual patient.

Inclusion Criteria: Patients 16 years old or younger at the time of injury, admitted to PICU for management of TBI with TBI-related pathology confirmed on CT or MRI and clinical requirement for invasive monitoring of ICP and ABP, will be eligible for recruitment. There are no exclusion criteria for the project.

Patient Recruitment and Consent: Each PTBI patient admitted to the recruiting PICU with invasive monitoring of ICP and ABP is considered for recruitment. The research team at each recruiting PICU identifies the patients and sets up high-resolution data collection to the project laptop under an anonymised ID as soon as possible after the patient has been commenced on invasive monitoring and stabilized on PICU. Physiological and clinical data is recorded under this unique study ID. As the acute phase of the project is purely observational and does not involve any therapeutic intervention or change in regular standard care, we take a deferred consent for acute data collection and sharing. This is important to obtain a bias-free sample and ensure capture of early

For peer review only

For peer review only - http://bmjopen.bmj.com/site/about/guidelines.xhtml

changes in intracranial physiology after head injury. When appropriate, the identified guardian/parent is informed and consented for data sharing and follow-up before the patient is discharged from the hospital. For patients who die before the consent can be obtained, the clinician in charge of patient's clinical care only (not study team) will access and anonymise data of the deceased patient under his/her clinical care; this anonymised data will then be provided to the study team.

Data Collection: ICP will be monitored with an intraparenchymal microsensor inserted into the right (predominately but can be left) frontal cortex and ABP will be monitored in the radial or femoral artery with a zero calibration at the level of the right atrium. The head end elevation is part of the standard international protocol for management for PTBI, and any deviations from this policy in individual cases will be recorded. Full resolution data of routinely monitored physiological parameters in managing PTBI [ICP, ABP, $ETCO_2$, SpO_2 and ECG] will be collected from bed-side monitors. Pulsatile signals [ICP, ABP and ECG] will be recorded with the maximum sampling frequency offered by the bed-side monitors, at least 100 Hz for invasive pressures, and at least 200Hz for ECG. This will be done using relevant, monitor type specific, data export protocols implemented in the study (ICM+) software running on a bed-side laptop. The same software will be later used for the retrospective analysis of all stored signals. The continuous data collection will be stopped when the patient either starts to wake up or dies and the invasive monitoring is stopped. Apart from general demographics and clinical data, the other data being collected are mechanism and details of injury, relevant past history, post resuscitation Glasgow coma scale, initial pupillary size and reactivity, pre-hospital hypoxia/ hypotension/ hyperthermia/ cardiopulmonary resuscitation, injury & disease severity scores (Pediatric Index of Mortality- PIM II score, Injury Severity Score- ISS), CT scan findings. We are also collecting: daily laboratory values (hemoglobin, glucose, sodium, lactate), PaO_2, $PaCO_2$ and FiO_2, PEEP, medical interventions for TBI (positioning, collar, temperature, CO_2, glucose, hypertonic saline or mannitol, sedation/paralysis, barbiturate coma, anti-seizure medication, use of vasoactive drugs to maintain blood pressure & surgical interventions (haematoma evacuation, elevation of depressed fracture, CSF diversion-external ventricular drainage, new brain imaging, any other relevant event.

Data Management and Monitoring: Each patient is allocated a unique study ID and all the collected data is anonymised to this ID. Appointed data manager is responsible for monitoring data transfer to Cambridge, for data verification, validation and repackaging, and storing it in the archive location on the server. Full ethical and

For peer review only - http://bmjopen.bmj.com/site/about/guidelines.xhtml

legal practice is being followed and database will be managed securely under the supervision of data custodian. The project is funded for five years (30th June 2023) at present and we will endeavor to get further funding to carry on the data collection and database further in the near future. The data will be stored on a secure server located on a private network behind a tight firewall at the sponsoring institute. We will use the already established system in use for similar larger adult project, CENTER-TBI (https://www.center-tbi.eu/). Briefly, all the physiological time series data collected in each participating site using ICM+ software are stripped of any identifiers and packaged into the HDF5 (https://www.hdfgroup.org/) based format. The file is then uploaded using the SSH data transfer protocol function of ICM+ to the Cambridge server using public/private key authentication and the username/password distributed in an encrypted format and installed on the ICM+ laptop. The data comes into 'upload' folder on that server and is then promptly moved to a staging folder, inaccessible via the SSH connection. The data is then scrutinized for potential problems, artifacts, stripped of any potential identifiers embedded into the annotations, stripped of the data recording date (the timestamps are recorded as relative to the date of ictus, not kept with the data file) and archived permanently. The original data is then removed. All analysis is subsequently done on that pre-processed data. The data is backed up daily, using incremental backup procedure.

Outcome Measures: The primary outcome will be measured by Glasgow Outcome Scale Extended Pediatric version (GOSE-Peds) at 12-months post-injury against the PRx.

The study will also include the following secondary outcome measures:

- GOSE-Peds at 6 months against the PRx
- Delta CPP (CPP deviation from CPPopt) against the GOSE-Peds at 6 & 12 months
- Rate of severe TBI-related surgical interventions undertaken during the index admission
- Mortality (30-day, 6 months, 12 months)
- Length of stay in hospital and rehabilitation

There is more detailed neuropsychology assessment at 12 months follow-up by using telephone-based interviews and postal questionnaires with parents by using the following tests (child versions will be used where available): BRIEF, PedsQL, SDQ, CCC, CASP and RCADS. In addition, with parental permission, school teacher will be contacted for SDQ and BRIEF with postal questionnaires.

Due to the covid-related impact on research, to circumvent the missed follow-ups for the recruited patients:

For peer review only - http://bmjopen.bmj.com/site/about/guidelines.xhtml

1. Where possible, site teams will carry out telephone interview with parents/legal guardian to ascertain child's neuro-developmental performance at the 1st anniversary of child's head injury for retrospective GOSE-Peds score at 12 months. There is precedence to this approach, especially in children where the developmental milestones/neurodevelopment are judged from memory based on how an individual child performed at different time-points in their developmental life (17-20).

2. If the child was seen in Acquired Brain Injury/Neurology/Neuro-psychology/Neurodevelopmental clinic at a specific site, the local research team will review the clinic letters/notes to ascertain child's outcome as close as possible to 12 months from the head injury.

Statistics

Power Calculation: The sample size has been calculated assuming a mean (± SD) PRx of 0.03 ± 0.13 in patients with favourable outcome and a mean (± SD) PRx of 0.10 ± 0.17 (SD) for patients with unfavourable outcome (from our previous work) (6). For a one-sided analysis, using a favourable/unfavourable ratio of 0.77, with 80% power and alpha error of 5%, and allowing for losses (protocol violations, withdrawal of consent, or loss of follow up) the study will require 135 patients. Recruiting 135 patients with severe TBI in 36 months should have been feasible as based on the national audits of PICU [PICAnet report 2015: 188, 210, 211 patients in 2013, 2014 and 2015 respectively with ICP devices from the 10 UK centres recruiting into the current study] and major trauma [TARN report on 2 years of severe injury in children (2013-2014): 1018 children in two years with severe head injury] with an estimated recruitment rate of 5-7 patients per site per year. On the basis of hospital episode statistics (HES) and data from the on-going local severe traumatic brain injury audit, approximately 5-10 patients with severe TBI are admitted in a medium-sized PICU each year.

However, due to the Covid-19 pandemic, the recruitment has been slow (pause of non-covid research projects, research staff re-deployment to clinical work and lockdowns leading to changes in trauma patterns in children), and the study has been extended until December 2024 in agreement with the sponsor and funding organisation.

Statistical Analysis: Continuous variables will be assessed for normality and expressed as mean (SD) or median (interquartile range), depending on the underlying distribution of the data. Categorical variables will be reported as counts and proportions. Differences in physiological values between survivors and non-survivors will be interrogated with the Mann-Whitney U-test for non-normally distributed continuous variables, Student t-test for normally distributed continuous variables and Fisher exact test for categorical variables. All tests will be two-tailed and unadjusted for multiple comparisons. Spearman rank correlation coefficient (ρ) will be used to see

For peer review only - http://bmjopen.bmj.com/site/about/guidelines.xhtml

For peer review only

correlations between variables. Identification of critical threshold values of PRx for predicting unfavorable outcome will be performed using iterative chi-square tests, as per the method described by Sorrentino et al (21). To account for any possible confounding effect of the differing periods of monitoring and day post admission on which monitoring starts on outcome associations, ordinal logistic regression analysis will be performed. The independent effect of each continuously monitored variable on GOSE-Peds will be assessed by including the length of monitoring and monitoring commencement day post admission as covariates in the regression models. Where appropriate PRx will be Fisher transformed to remove the <-1;1> constrain and ensure normalization of its distribution. The effect of time on PRx will be assessed using repeated-measures analysis of variance with each patient treated as a random effect.

For peer review only

<u>Participant Confidentiality and data protection</u>: The data collection part of this research study is limited to accessing, collecting and analyzing information that is monitored and placed in the medical record as part of the child's standard care while being treated for severe TBI in PICU. Our plan to protect patient-subject identifiers from improper use and disclosure is consistent with our plans to protect the overall data of the study. Specifically, access to patient-subject identifiers will be limited to study personnel that require its use for clinical reasons. The anonymised data will be used by the investigators and research staff listed on this application which will help ensure that there is no improper use or disclosure. Secondly, a limited amount of personal information (specifically, only the month and year of birth which is essential to establishing the age of the subject) will be collected. Third, all patient-subject identifiers will be kept in a locked cabinet within the recruiting hospital, which can only be accessed by the study staff on the delegation log. Fourth, all subjects within the study will be given a unique, de-identified study ID and this study ID will be utilized for importing data to the study database. We believe that our plan adequately ensures that patient-subject identifiers will be protected from improper use and disclosure. There will be no patient identifiable information shared outside the respective clinical teams looking after each patient. Within each individual centre, the patient identifiable information will not be seen/shared with anyone who does not access it otherwise (outside the clinical teams). Within the centres, the consent forms and the data collection will be stored in a secure locked cupboard within a secure locked room. The individual centres will be instructed to save the data for five years (to ensure data auditing and clearance), after which time the collected identifiable data will be destroyed).

The project is to be carried out in conformation with the spirit and the letter of the declaration of Helsinki, and in accord with the ICH Good Clinical Practice Guidelines. The application, the database management and

For peer review only - http://bmjopen.bmj.com/site/about/guidelines.xhtml

monitoring have full support from the patient safety department at sponsoring institute, and the protocol has been approved by them. The entire project will be conducted under close scrutiny of data custodian and the patient safety department.

Ethics Approval & Regulatory Considerations: Ethical approval was provided by Health Research Authority, South West- Central Bristol Research Ethics Committee (Ref: 18/SW/0053). As the project has approval for research database, it did not require site specific ethics. The project was reviewed and approved at each participating site before starting recruitment. The study protocol, patient information sheet, consent forms, and other study related documents were reviewed and approved by the REC with regards to their scientific content and compliance with applicable research regulations involving human subjects. The current study protocol is v5 dated 28/08/2018. The study is registered at ClinicalTrails.gov with ID- NCT05688462.

Dissemination: The results will be published in peer-reviewed journals and presented at relevant national and international conferences and seminars.

Acknowledgements: The authors thank Ricardo G Branco, Joseph Donnelly and Matthew Garnett for their valuable contribution to the study design. The authors also thank all the research and clinical staff at the participating sites: Birmingham Children's Hospital, Great Ormond Street Hospital, Leeds General Hospital, Manchester Children's Hospital, Queen's Medical Centre Nottingham, John Radcliffe Hospital Oxford, Royal London Hospital, Sheffield Children's Hospital and Southampton Children's Hospital.

Contributors: SA is the chief investigator, DW and ED are study nurses, MP and MC have been data coordinators and research associates for the study providing technical support, SW has contributed to neuro-developmental testing. PS, MC, AY, AM and PH are trial co-applicants. All the authors have read and approved the final version of this manuscript. SA, PS, MC, PH are part of the study steering committee.

Funding: This study is funded by Action Medical Research for Children's Charity, UK (grant number-GN2609). Cambridge University Hospitals is the study sponsor (reference: A094693, contact person: Michelle Ellerbeck- michelle.ellerbeck@nhs.net).

For peer review only - http://bmjopen.bmj.com/site/about/guidelines.xhtml

Conflict of interest: ICM+ is a software licensed by Cambridge Enterprise Ltd. Peter Smielewski and Marek Czosnyka have a financial interest in a part of licensing fee; the licensing fee was waivered for this study. There are no additional patents, products in development or marketed products to declare.

Data Access: The final database will be saved on a non-public secure repository, the access to the data will be available on request.

Legend for Figure 1: PRx and CPPopt calculation in real-time in PTBI

PRx and CPPopt calculation in real time in a patient recruited to STARSHIP in an 8-hour recording window. Top panel displays ABP, followed by ICP, and then CPP (CPPopt in the light grey line and lower limit of autoregulation in dark grey line). The bottom panel shows calculation of CPPopt. CPPopt is continuously calculated using a 5-min median CPP time trend alongside PRx. These PRx values are divided and averaged into CPP bins spanning 5 mm Hg. An automatic curve fitting method is applied to the binned data to determine the CPP value with the lowest associated PRx value. (Author's own work)

PRx- cerebrovascular pressure reactivity index, CPPopt- optimum cerebral perfusion pressure, PTBI- Pediatric Traumatic brain injury, ABP- arterial blood pressure, ICP- intracranial pressure, CPP- cerebral perfusion pressure.

For peer review only

References:

1. Faul M, Xu L, Wald MM, Coronado VG. Traumatic brain injury in the United States: emergency department visits, hospitalizations, and deaths. Atlanta (GA): Centers for Disease Control and Prevention, National Center for Injury Prevention and Control; 2010.

2. Dewan MC MN, Wellons III C, Bonfield CM. Epidemiology of Global Pediatric Traumatic Brain Injury: Qualitative Review. World Neurosurg 2016;91:497-509.

3. Langlois JA, Rutland-Brown W, Thomas KE. Traumatic Brain Injury in the United States: Emergency Department Visits, Hospitalizations, and Deaths. Atlanta, GA: Centers for Disease Control and Prevention; 2004.

4. Bayreuther J, Wagener S, Woodford M, et al. Paediatric trauma: Injury pattern and mortality in the UK. Arch Dis Child Educ Pract Ed 2009;94:37-41

5. Kochanek PM, Tasker RC, Nancy C, et al,. Guidelines for the management of pediatric severe traumatic brain, third edition: update of the Brain Trauma Foundation Guidelines. Pediatr Crit Care Med. 2019;20:S1-82.

6. Young AM, Donnelly J, Czosnyka M, et al. Continuous multimodality in children after traumatic brain injury-preliminary experience. PLoS One 2016;11:e0148817. doi: 10.1371/journal.pone.0148817.

7. Lewis PM, Czosnyka M, Carter BG, et al. Cerebrovascular pressure reactivity in children with traumatic brain injury. Pediatr Crit Care Med 2015;16:739-49.

8. Brady KM, Shaffner DH, Lee JK, et al. Continuous monitoring of cerebrovascular pressure reactivity after traumatic brain injury in children. Pediatrics 2009;124:e1205-12. doi: 10.1542/peds.2009-0550.

9. Aries MJ, Czosnyka M, Budohoski KP, et al, Continuous determination of optimal cerebral perfusion pressure in traumatic brain injury. Crit Care Med. 2012;40:2456-63.

10. Steiner LA, Czosnyka M, Piechnik SK, et al. Continuous monitoring of cerebrovascular pressure reactivity allows determination of optimal cerebral perfusion pressure in patients with traumatic brain injury. Crit Care Med. 2002;30:733–8.

11. Lang EW, Chesnut RM: Intracranial pressure and cerebral perfusion pressure in severe head injury. New Horiz 1995; 3:400–409.

12. Paulson OB, Strandgaard S, Edvinsson L: Cerebral autoregulation. Cerebrovasc Brain Metab Rev 1990; 2:161–192.

13. Le Roux P, Menon DK, Citerio G, et al,. The International Multidisciplinary Consensus Conference on Multimodality Monitoring in Neurocritical Care: Evidentiary Tables: A Statement for Healthcare Professionals from the Neurocritical Care Society and the European Society of Intensive Care Medicine. Neurocrit Care. 2014;21:S297-361.

14. Czosnyka M, Smielewski P, Kirkpatrick P, Laing RJ, Menon D, Pickard JD. Continuous assessment of the cerebral vasomotor reactivity in head injury. Neurosurgery. 1997;41:11–7.

15. Johnson U, Lewen A, Ronne-Engstrom E, et al. Should the neuro-intensive care management of traumatic brain injury patients be individualized according to autoregulation status and injury subtype? Neurocrit Care 2014;21:259-65.

16. Jaeger M, Lang EW. Cerebrovascular pressure reactivity and cerebral oxygen regulation after severe head injury. Neurocrit Care 2013;19:69-73.

17. Bejerot S, Lugnegard T. Retrospective parental assessment of childhood neurodevelopmental problems: the use of the Five to Fifteen questionnaire in adults. B J Psych Open 2019;5:1-5. doi:10.1192/bjo.2019.30.

For peer review only - http://bmjopen.bmj.com/site/about/guidelines.xhtml

18. Lord C, Rutter M, Couteur AL. Autism diagnostic interview- revised: A revised version of a diagnostic interview for caregivers of individuals with possible pervasive developmental disorders. J Autism Developmental Disorders 1994;24:659-685.

19. Fombonne E. Diagnostic assessment in a sample of autistic and developmentally impaired adolescents. J Autism Developmental Disorders 1992;22:563-581.

20. Hirvikoski T, Jokinen SLJ, Renhorn E, Trillingsgaard A, Kadesjo B, Borg J, The FTF working group. Using the five to fifteen collateral informant questionnaire for retrospective assessment of childhood symptoms in adults with and without autism or ADHD. Eur Child Adol Psych 2021;30:1367-1381.

21. Sorrentino E, Diedler J, Kasprowicz M, Budohoski KP, Haubrich C, Smielewski P, Outtrim JG, Manktelow A, Hutchinson PJ, Pickard JD, Menon DK, Czosnyka M. Critical thresholds for cerebrovascular reactivity after traumatic brain injury. Neurocrit Care 2012;16:258-66

For peer review only

For peer review only - http://bmjopen.bmj.com/site/about/guidelines.xhtml

Figure 1:

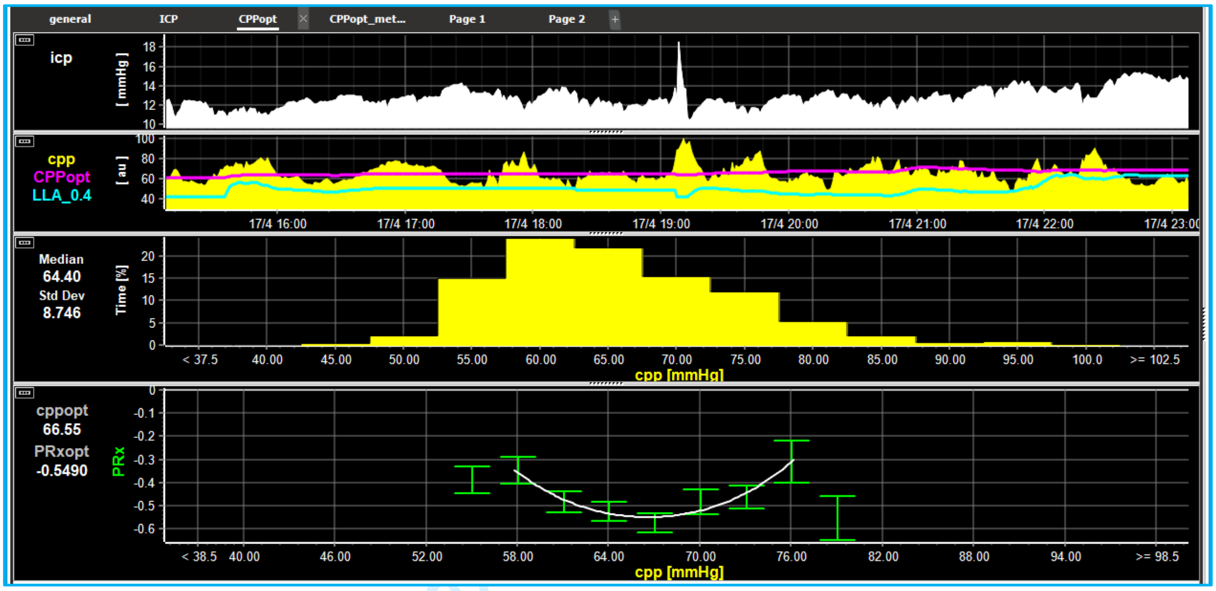

For peer review only

Reporting checklist for protocol of a clinical trial.

Based on the SPIRIT guidelines.

Instructions to authors

Complete this checklist by entering the page numbers from your manuscript where readers will find each of the items listed below.

Your article may not currently address all the items on the checklist. Please modify your text to include the missing information. If you are certain that an item does not apply, please write "n/a" and provide a short explanation.

Upload your completed checklist as an extra file when you submit to a journal.

In your methods section, say that you used the SPIRITreporting guidelines, and cite them as:

Chan A-W, Tetzlaff JM, Gøtzsche PC, Altman DG, Mann H, Berlin J, Dickersin K, Hróbjartsson A, Schulz KF, Parulekar WR, Krleža-Jerić K, Laupacis A, Moher D. SPIRIT 2013 Explanation and Elaboration: Guidance for protocols of clinical trials. BMJ. 2013;346:e7586

		Reporting Item	Page Number
**Administrative information**			
Title	#1	Descriptive title identifying the study design, population, interventions, and, if applicable, trial acronym	1
Trial registration	#2a	Trial identifier and registry name. If not yet registered, name of intended registry	1
Trial registration: data set	#2b	All items from the World Health Organization Trial Registration Data Set	
Protocol version	#3	Date and version identifier	9
Funding	#4	Sources and types of financial, material, and other support	11
Roles and responsibilities: contributorship	#5a	Names, affiliations, and roles of protocol contributors	1, 11

For peer review only

Roles and responsibilities: sponsor contact information	#5b	Name and contact information for the trial sponsor	11
Roles and responsibilities: sponsor and funder	#5c	Role of study sponsor and funders, if any, in study design; collection, management, analysis, and interpretation of data; writing of the report; and the decision to submit the report for publication, including whether they will have ultimate authority over any of these activities	11
Roles and responsibilities: committees	#5d	Composition, roles, and responsibilities of the coordinating centre, steering committee, endpoint adjudication committee, data management team, and other individuals or groups overseeing the trial, if applicable (see Item 21a for data monitoring committee)	11
**Introduction**			
Background and rationale	#6a	Description of research question and justification for undertaking the trial, including summary of relevant studies (published and unpublished) examining benefits and harms for each intervention	3
Background and rationale: choice of comparators	#6b	Explanation for choice of comparators	3
Objectives	#7	Specific objectives or hypotheses	4
Trial design	#8	Description of trial design including type of trial (eg, parallel group, crossover, factorial, single group), allocation ratio, and framework (eg, superiority, equivalence, non-inferiority, exploratory)	4-7
**Methods: Participants, interventions, and outcomes**			
Study setting	#9	Description of study settings (eg, community clinic, academic hospital) and list of countries where data will be collected. Reference to where list of study sites can be obtained	5

For peer review only

Eligibility criteria	#10	Inclusion and exclusion criteria for participants. If applicable, eligibility criteria for study centres and individuals who will perform the interventions (eg, surgeons, psychotherapists)	5
Interventions: description	#11a	Interventions for each group with sufficient detail to allow replication, including how and when they will be administered	5-6
Interventions: modifications	#11b	Criteria for discontinuing or modifying allocated interventions for a given trial participant (eg, drug dose change in response to harms, participant request, or improving / worsening disease)	NA
Interventions: adherance	#11c	Strategies to improve adherence to intervention protocols, and any procedures for monitoring adherence (eg, drug tablet return; laboratory tests)	NA
Interventions: concomitant care	#11d	Relevant concomitant care and interventions that are permitted or prohibited during the trial	NA
Outcomes	#12	Primary, secondary, and other outcomes, including the specific measurement variable (eg, systolic blood pressure), analysis metric (eg, change from baseline, final value, time to event), method of aggregation (eg, median, proportion), and time point for each outcome. Explanation of the clinical relevance of chosen efficacy and harm outcomes is strongly recommended	7
Participant timeline	#13	Time schedule of enrolment, interventions (including any run-ins and washouts), assessments, and visits for participants. A schematic diagram is highly recommended (see Figure)	NA
Sample size	#14	Estimated number of participants needed to achieve study objectives and how it was determined, including clinical and statistical assumptions supporting any sample size calculations	8
Recruitment	#15	Strategies for achieving adequate participant enrolment to reach target sample size	5
**Methods: Assignment of interventions (for controlled trials)**			
Allocation: sequence generation	#16a	Method of generating the allocation sequence (eg, computer-generated random numbers), and list of any factors for stratification. To reduce predictability of a random sequence, details of any planned restriction (eg, blocking) should be	NA

For peer review only

		provided in a separate document that is unavailable to those who enrol participants or assign interventions	
Allocation concealment mechanism	#16b	Mechanism of implementing the allocation sequence (eg, central telephone; sequentially numbered, opaque, sealed envelopes), describing any steps to conceal the sequence until interventions are assigned	NA
Allocation: implementation	#16c	Who will generate the allocation sequence, who will enrol participants, and who will assign participants to interventions	NA
Blinding (masking)	#17a	Who will be blinded after assignment to interventions (eg, trial participants, care providers, outcome assessors, data analysts), and how	NA
Blinding (masking): emergency unblinding	#17b	If blinded, circumstances under which unblinding is permissible, and procedure for revealing a participant's allocated intervention during the trial	NA
**Methods: Data collection, management, and analysis**			
Data collection plan	#18a	Plans for assessment and collection of outcome, baseline, and other trial data, including any related processes to promote data quality (eg, duplicate measurements, training of assessors) and a description of study instruments (eg, questionnaires, laboratory tests) along with their reliability and validity, if known. Reference to where data collection forms can be found, if not in the protocol	5-7
Data collection plan: retention	#18b	Plans to promote participant retention and complete follow-up, including list of any outcome data to be collected for participants who discontinue or deviate from intervention protocols	5-7
Data management	#19	Plans for data entry, coding, security, and storage, including any related processes to promote data quality (eg, double data entry; range checks for data values). Reference to where details of data management procedures can be found, if not in the protocol	6-7
Statistics: outcomes	#20a	Statistical methods for analysing primary and secondary outcomes. Reference to where other details of the statistical analysis plan can be found, if not in the protocol	7-8

For peer review only

Statistics: additional analyses	#20b	Methods for any additional analyses (eg, subgroup and adjusted analyses)	8
Statistics: analysis population and missing data	#20c	Definition of analysis population relating to protocol non-adherence (eg, as randomised analysis), and any statistical methods to handle missing data (eg, multiple imputation)	NA
**Methods: Monitoring**			
Data monitoring: formal committee	#21a	Composition of data monitoring committee (DMC); summary of its role and reporting structure; statement of whether it is independent from the sponsor and competing interests; and reference to where further details about its charter can be found, if not in the protocol. Alternatively, an explanation of why a DMC is not needed	11
Data monitoring: interim analysis	#21b	Description of any interim analyses and stopping guidelines, including who will have access to these interim results and make the final decision to terminate the trial	NA
Harms	#22	Plans for collecting, assessing, reporting, and managing solicited and spontaneously reported adverse events and other unintended effects of trial interventions or trial conduct	NA
Auditing	#23	Frequency and procedures for auditing trial conduct, if any, and whether the process will be independent from investigators and the sponsor	NA
**Ethics and dissemination**			
Research ethics approval	#24	Plans for seeking research ethics committee / institutional review board (REC / IRB) approval	9
Protocol amendments	#25	Plans for communicating important protocol modifications (eg, changes to eligibility criteria, outcomes, analyses) to relevant parties (eg, investigators, REC / IRBs, trial participants, trial registries, journals, regulators)	7
Consent or assent	#26a	Who will obtain informed consent or assent from potential trial participants or authorised surrogates, and how (see Item 32)	5

For peer review only

Consent or assent: ancillary studies	#26b	Additional consent provisions for collection and use of participant data and biological specimens in ancillary studies, if applicable	NA
Confidentiality	#27	How personal information about potential and enrolled participants will be collected, shared, and maintained in order to protect confidentiality before, during, and after the trial	9
Declaration of interests	#28	Financial and other competing interests for principal investigators for the overall trial and each study site	11
Data access	#29	Statement of who will have access to the final trial dataset, and disclosure of contractual agreements that limit such access for investigators	11
Ancillary and post trial care	#30	Provisions, if any, for ancillary and post-trial care, and for compensation to those who suffer harm from trial participation	
Dissemination policy: trial results	#31a	Plans for investigators and sponsor to communicate trial results to participants, healthcare professionals, the public, and other relevant groups (eg, via publication, reporting in results databases, or other data sharing arrangements), including any publication restrictions	10
Dissemination policy: authorship	#31b	Authorship eligibility guidelines and any intended use of professional writers	
Dissemination policy: reproducible research	#31c	Plans, if any, for granting public access to the full protocol, participant-level dataset, and statistical code	
**Appendices**			
Informed consent materials	#32	Model consent form and other related documentation given to participants and authorised surrogates	
Biological specimens	#33	Plans for collection, laboratory evaluation, and storage of biological specimens for genetic or molecular analysis in the current trial and for future use in ancillary studies, if applicable	

None The SPIRIT Explanation and Elaboration paper is distributed under the terms of the Creative Commons Attribution License CC-BY-NC. This checklist can be completed online using https://www.goodreports.org/, a tool made by the EQUATOR Network in collaboration with Penelope.ai

For peer review only
