# Supplementary material for: Studying Trends of Auto-Regulation in Severe Head Injury in Paediatrics (STARSHIP): protocol to study cerebral autoregulation in a prospective multicentre observational research database study
Source: BMJ Open. 2023 Mar 10;13(3):e071800. doi: 10.1136/bmjopen-2023-071800 (PMC10008199; doi:10.1136/bmjopen-2023-071800)
Supplement: Reviewer comments [file bmjopen-2023-071800.reviewer_comments.pdf]

PEER REVIEW HISTORY

BMJ Open publishes all reviews undertaken for accepted manuscripts. Reviewers are asked to complete a checklist review form (**http://bmjopen.bmj.com/site/about/resources/checklist.pdf**) and are provided with free text boxes to elaborate on their assessment. These free text comments are reproduced below.

ARTICLE DETAILS

**TITLE (PROVISIONAL)**	Studying Trends of Auto-Regulation in Severe Head Injury in Paediatrics (STARSHIP): Protocol to study cerebral autoregulation in a prospective multicentre observational research database study.
**AUTHORS**	Agrawal, Shruti; Placek, Michal M.; White, Deborah; Daubney, Esther; Cabeleira, Manuel; Smielewski, Peter; Czosnyka, Marek; Young, Adam; Watson, Suzanna; Maw, Anna; Hutchinson, Peter

This article was not externally reviewed at BMJ Open. Protocols that have been independently assessed prior to submission to BMJ Open are usually fast-tracked to publication on the grounds that further substantial changes will not be possible. This independent assessment will usually be external, independent review for both a substantial grant award from a non-commercial or government funder and ethics approval.
